# Methodological improvements are needed in network meta analyses of antidiabetic drugs for type 2 diabetes mellitus

**DOI:** 10.3389/fendo.2026.1734108

**Published:** 2026-02-25

**Authors:** Anna Dai, Yang Liu, Jiali Yin, Qianqian Wang, Maoyi Yang, Xiaoli Ji, Zhipeng Hu

**Affiliations:** 1 Clinical Medical College, Chengdu University of Traditional Chinese Medicine, Chengdu, Sichuan, China; 2 Hospital of Chengdu University of Traditional Chinese Medicine, Chengdu, China

**Keywords:** assessment of multiple systematic reviews 2, methodological quality, network meta-analyses, reporting quality, systematic reviews, type 2 diabetes mellitus

## Abstract

**Introduction:**

Numerous network meta-analyses have compared the efficacy of antidiabetic drugs for type 2 diabetes mellitus. However, a systematic assessment of their methodological and reporting quality remains lacking.

**Methods:**

We searched the PubMed, Embase, and Cochrane Library databases for relevant studies from inception to July 21, 2025 for published network meta-analyses that compares the efficacy of antidiabetic drugs for type 2 diabetes mellitus. The methodological and reporting quality of included studies was assessed by the Assessment of Multiple Systematic Reviews 2 checklist and the PRISMA-NMA checklist. Regression analyses were performed to identify factors potentially associated with the methodological quality of the included studies.

**Results:**

A total of 185 NMAs were included. Over half of the studies (58.38%) originated from developing countries. Most studies focused on comparison of multiple antidiabetic drugs (122, 65.95%), and the majority used Bayesian methods (98, 52.97%). The overall methodological quality was low, with only one study rated as high quality. Critical items of AMSTAR 2 were poorly reported, with only 2.70% of studies adequately reporting item 7 and 58.38% reporting item 2. Significant associations were observed between study quality and analytical framework, outcome, economic development level, journal impact factor, and protocol registration status.

**Conclusion:**

Network meta-analyses that compares antidiabetic drugs for T2DM exhibit substantial methodological limitations and advancements in methodology are critically needed in future research.

## 1 Introduction

Type 2 diabetes mellitus (T2DM) is a major global endocrine and metabolic disorder, primarily characterized by defective insulin secretion and insulin resistance (1). According to the 11th edition of the International Diabetes Federation (IDF) Diabetes Atlas, an estimated 589 million adults aged 20–79 were living with diabetes globally in 2024, representing 11.1% of this age demographic (2). T2DM accounts for approximately 90–95% of all diabetes cases (3). Affected individuals face a significantly elevated risk of developing severe microvascular and macrovascular complications (1, 4, 5). The associated global healthcare expenditure amounts to tens of billions of US dollars annually, imposing a substantial economic strain on healthcare systems worldwide (6).

According to the 2025 American Diabetes Association (ADA) Standards of Medical Care in Diabetes, antidiabetic drugs for T2DM include non-insulin medication: metformin, sodium-glucose cotransporter-2 inhibitors (SGLT2i), glucagon-like peptide-1 receptor agonists (GLP-1 RAs), dual glucose-dependent insulinotropic polypeptide and GLP-1 receptor agonists (GIP/GLP-1 RAs), dipeptidyl peptidase-4 inhibitors (DPP-4i), thiazolidinediones (TZDs, e.g., pioglitazone), and sulfonylureas (SU), as well as insulin preparations, including human insulin and insulin analogs (7).

Systematic reviews (SRs) constitute a rigorous research methodology designed to comprehensively identify, appraise, and synthesize all pertinent evidence pertaining to a predefined clinical question according to an *a priori* protocol (8). This process facilitates the systematic collation and critical appraisal of existing clinical research, thereby generating robust evidence to inform healthcare decisions. A pivotal step in conducting an SR involves the determination of whether numerical data from the included studies can be statistically pooled. When these studies demonstrate sufficient homogeneity in their designs, populations, and outcomes, meta-analysis (MA) can be implemented as a statistical technique to quantitatively combine results from multiple independent investigations. Specifically, MA allows for the calculation of a pooled effect estimate (and its confidence interval) that summarizes the comparative efficacy of an experimental intervention against a control (9). Nevertheless, a fundamental limitation of traditional SRs and MAs is their confinement to synthesizing evidence from studies involving direct comparisons between only two interventions. In clinical practice, however, therapeutic strategies are often multifaceted, necessitating comparisons across a range of options. The prevalent paucity of head-to-head trials comparing all relevant interventions directly thus underscores a critical flaw of conventional evidence synthesis in facilitating comprehensive comparative assessments.

Network meta-analysis (NMA), an extension of traditional pairwise meta-analysis, utilizes both direct and indirect evidence to simultaneously compare the efficacy of three or more interventions (10). This approach overcomes a key limitation of conventional systematic reviews, which cannot evaluate multiple interventions within a single analytical framework (11, 12). Empirical evidence demonstrates that NMA provides more precise effect estimates compared to individual direct or indirect comparisons (13, 14). Owing to this comparative advantage, numerous NMAs evaluating hypoglycemic agents for T2DM have been published (15, 16). However, NMA shares methodological limitations with traditional pairwise meta-analysis, as its conclusions are contingent upon the quality of its execution (17). Findings from methodologically flawed NMAs pose a significant risk of misleading clinical practice. Consequently, a critical appraisal of the methodological quality of existing NMAs is imperative to identify current shortcomings and inform improvements in future research.

Assessment of Multiple Systematic Reviews 2 (AMSTAR 2) is currently the most critical assessment tool for systematic reviews, primarily applied to randomized and non-randomized studies of healthcare interventions (18). Owing to the methodological similarities between network meta-analyses, systematic reviews and traditional meta-analyses, the AMSTAR 2 tool is also widely used to evaluate the methodological quality of NMAs (19, 20, p.139). Meanwhile, the reporting of NMAs should also adhere to the PRISMA-NMA statement (21, 22).

Currently, a comprehensive methodological assessment is lacking for the numerous published network meta-analyses comparing the efficacy of antidiabetic drugs for T2DM, which may lead to serious consequences. In clinical practice, methodological weaknesses in network meta-analyses may not only affect statistical inference but also carry important clinical consequences. Flawed evidence synthesis can lead to misleading treatment rankings (23), exaggerated or attenuated estimates of comparative efficacy, and premature assumptions regarding class effects (24). In several therapeutic domains, poorly conducted NMAs have been shown to influence the interpretation of benefit–risk trade-offs and, in some instances, to contribute indirectly to inappropriate therapeutic recommendations in clinical guidance (25, 26). Therefore, improving the methodological rigor of NMAs is essential not only from a research-quality perspective but also for safeguarding the integrity of evidence-based clinical decision-making. On one hand, study results that have not undergone rigorous quality assessment can compromise the integrity of the evidence base, ultimately misleading clinical guidelines and resulting in erroneous decision-making by clinicians. On the other hand, unaddressed methodological flaws often lead to the replication of low-quality studies in subsequent research, thereby wasting scarce scientific resources. Therefore, this study aims to evaluate and compare the methodological quality of network meta-analyses focusing on the efficacy comparison of antidiabetic drugs for T2DM, in order to provide a reference for conducting high-quality studies on the efficacy comparison of these drugs in the future.

## 2 Methods

The methods for this study are in accordance with “The PRISMA 2020 statement: An updated guideline for reporting systematic reviews.” (27).

### 2.1 Search strategy

We searched the PubMed, Embase, and Cochrane Library databases for relevant studies from inception to July 21, 2025. The search strategies for each database are shown in **Supplementary Material 1**.

### 2.2 Inclusion and exclusion criteria

Inclusion criteria: (1) Population: Adult patients diagnosed with type 2 diabetes mellitus (T2DM); (2) Intervention: Pharmacological interventions for T2DM as defined by the 2025 ADA Standards of Care, including: metformin, sodium-glucose cotransporter-2 inhibitors (SGLT2i), glucagon-like peptide-1 receptor agonists (GLP-1 RAs), dual glucose-dependent insulinotropic polypeptide and GLP-1 receptor agonists (GIP/GLP-1 RAs), dipeptidyl peptidase-4 inhibitors (DPP-4i), thiazolidinediones (TZDs, e.g., pioglitazone), and sulfonylureas (SU), human insulin, and insulin analogs; (3) Studies were designed as network meta-analyses (NMAs).

Exclusion criteria: (1) Population: Studies focusing on the treatment of diabetic complications (e.g., diabetic nephropathy) using non-antidiabetic medications, such as angiotensin-converting enzyme inhibitors (ACEIs), as these fall outside the scope of antidiabetic drugs specified in our protocol; (2) Interventions: Studies investigating Traditional Chinese Medicine, Chinese patent medicines, dietary supplements, fish oil, or probiotics, as these are not classified as core antidiabetic drugs per our predefined standards; (3) NMAs that did not perform a quantitative synthesis of data, as these cannot be adequately assessed using our methodological quality appraisal tools; (4) Studies not indexed in the Science Citation Index, as this review aims to synthesize evidence from analyses with established international recognition and influence; (5) Conference abstracts, posters, or studies for which only an abstract was available, due to insufficient methodological detail for comprehensive assessment.

### 2.3 Study selection

The study selection process was conducted according to the pre-specified protocol. Initially, all identified studies were imported into ZOTERO 7.0.26 reference management software for duplicate removal. Two reviewers (Dai and Liu) independently screened the titles and abstracts of the remaining records to exclude those clearly not meeting the inclusion criteria. The potentially eligible studies were subsequently retrieved for detailed assessment. When full texts were unavailable, the corresponding authors were contacted to obtain them. The full texts were then thoroughly reviewed to finalize study inclusion, with reasons for exclusion being documented. The selection process was summarized using a PRISMA flow diagram, and a list of excluded studies was provided in the **Supplementary Materials**. Any disagreements between the two reviewers were resolved through consultation with a third reviewer (Hu).

### 2.4 Data extraction

Data extraction was independently performed by two reviewers (Dai and Liu) using a predesigned data collection form. The following data were extracted from each included study into Microsoft Excel 16.101: number of authors, publication year, country of the first author, continent of the country, economic development level, journal impact factor, Journal Citation Reports (JCR) category, number of primary studies included, network meta-analysis methodology, study design(s), interventions, reporting of funding sources, and protocol publication status. Disagreements were resolved by a third reviewer (Hu).

### 2.5 Quality assessment

The methodological quality of the included studies was using the AMSTAR 2 tool (18). This tool is specifically designed for the critical appraisal of systematic reviews of healthcare interventions and comprises 16 items, 7 of which are considered critical. Each item was judged as “Yes”, “partial Yes”, or “No” based on the specific methodological details of each study. Subsequently, the overall grade of each study was categorized into one of four grades: “High”, “Moderate”, “Low”, or “ Critically low”. The detailed assessment criteria are provided in the **Supplementary Material 2**. The methodological quality assessment was performed using the AMSTAR 2 online tool (https://amstar.ca/Amstar_Checklist.php). This tool automatically generates an overall rating upon completion of all item assessments.

The reporting quality of the included studies was using the PRISMA-NMA statement, which comprises a checklist of 32 items (22). To evaluate the degree of compliance with the PRISMA-NMA statement, each item was assigned one of the following responses: “yes” for full compliance; “partial yes” for partial compliance; and “no” for noncompliance. Responses were scored as “1” point for each “yes,” “0.5” points for each “partial yes, “ and “0” points for each “no” (28, 29). The scores for all items were summed to derive a total quality score. Based on the total score, studies were categorized into three groups: low (up to the 25th percentile), moderate (the interquartile range), and high (the 75th percentile and above) (30). Additionally, for each item, percentage scores were arbitrarily classified as: good (>90%), medium (50%–90%), and poor (<50%) (31).

The methodological and reporting quality of the included studies was assessed independently by two reviewers (Dai and Liu). Disagreements were resolved by a third reviewer (Hu).

### 2.6 Data analysis

Data were analyzed using descriptive and inferential statistical methods. Categorical variables, including economic development level (developed/developing countries), continent, publication year (Up to and including 2017/After 2017, based on AMSTAR 2 publication date), JCR category (Q1–Q4, classified according to the JCR status at the time of this study), network meta-analysis methodology (Bayesian, frequentist, both, or unclear) (32), study design(s) (randomized clinical trials (RCTs) and non-RCTs, or mixed), interventions (DPP-4is, GLP-1 RAs, Insulin, SGLT2is, and Comparison of multiple antidiabetic drugs), outcomes (Glycemic, Cardiovascular, Kidney, Cardiovascular and kidney, Safety outcomes, and Other outcomes), reporting quality (high, moderate, low),reporting of funding sources (reported/unreported), and protocol publication status (pre-registered/not pre-registered), are presented as frequencies and percentages. Continuous variables—including journal impact factor, number of primary studies included, and number of authors—were described using medians (interquartile ranges) due to non-normal distribution. Group comparisons were performed using the chi-square test for categorical variables and the Kruskal–Wallis test for continuous variables. We further examined whether methodological quality differed across studies stratified by type of outcome, drug class, or analytical framework.

A binary logistic regression analysis was performed using R Studio version 4.3.1 to identify factors associated with methodological quality. The dependent variable was study quality, dichotomized into “Low” and “ Critically low” categories. Independent variables included economic development level (binary, with developing countries as reference), publication year (binary, with pre-AMSTAR 2 publication as reference), JCR category (binary, with Q3/Q4 as reference), protocol publication status (binary, with non-registered as reference), along with journal impact factor, number of primary studies included, and number of authors as continuous variables. The statistical significance was set at *P* < 0.05. All tables in this study were performed using Microsoft Word 16.101, while all figures were generated using Origin 2024.

Prior to performing multivariable logistic regression analysis, we assessed multicollinearity among the independent variables. Diagnostic measures, including the variance inflation factor (VIF) and tolerance, were calculated to evaluate potential collinearity. Generally, a VIF value exceeding 5 or 10 is considered indicative of substantial multicollinearity (33). In the present study, all independent variables exhibited VIF values ranging from 1.08 to 1.96, all well below the threshold of 5, suggesting no substantial multicollinearity concerns. Specific details are shown on **Table 1**.

**Table 1:** Statistical results of multicollinearity.

Variable	Vif results	Tolerance
Economic development level	1.075464212	0.929831034
Publication year	1.165577816	0.857943576
IF	1.960048522	0.510191451
JCR	1.090338471	0.917146397
Included studies	1.317665291	0.75891807
Number of authors	1.397248359	0.715692378
Protocol publication	1.858047605	0.538199343

## 3 Results

### 3.1 Study selection

**Figure 1:**
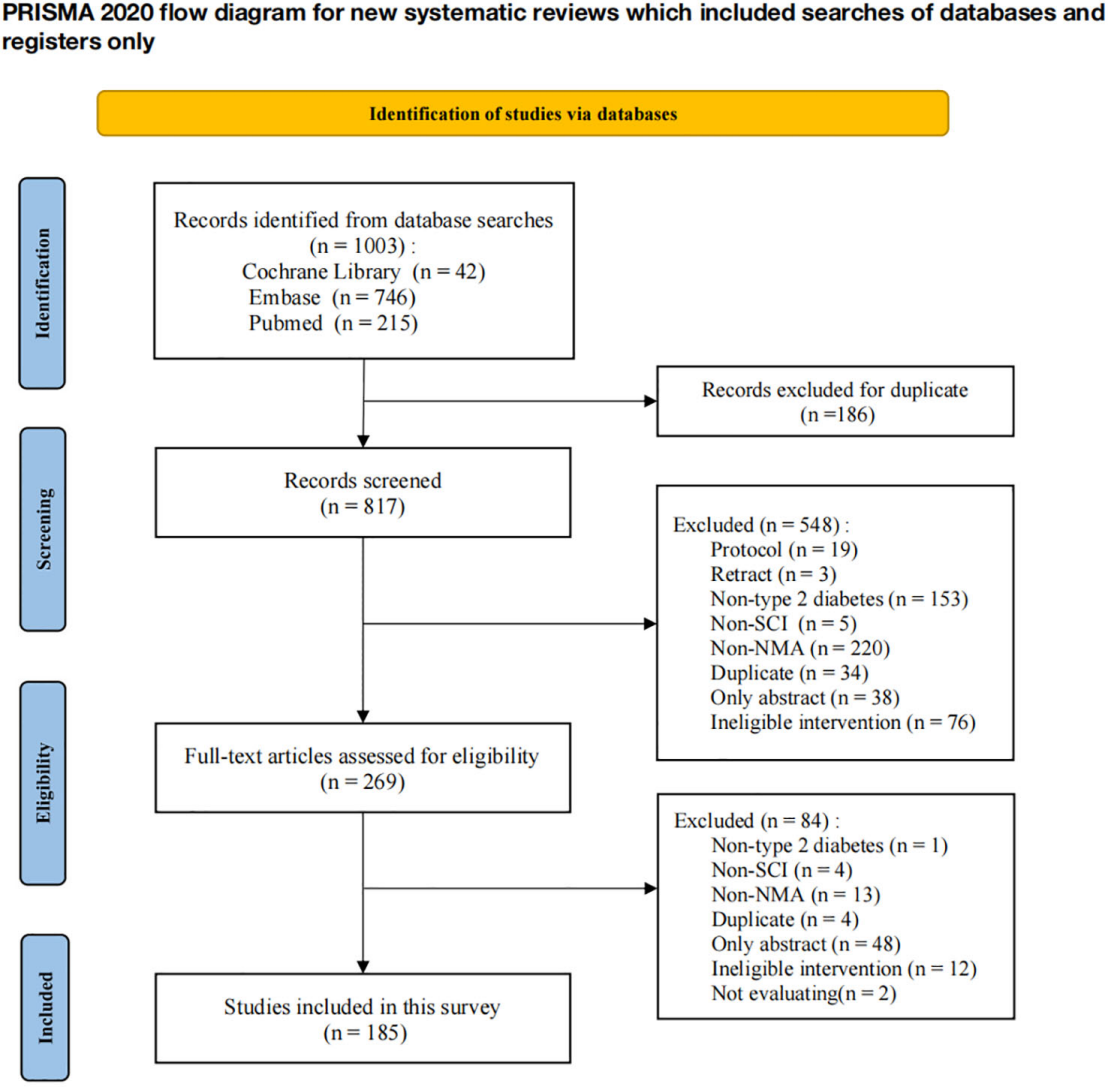
Flow diagram of the studies screening.

The study selection process is detailed in **Figure 1**. A total of 1,003 records were identified through database searching: 215 from PubMed, 746 from EMBASE, and 42 from the Cochrane Library. After removing 186 duplicates, 817 records underwent title and abstract screening, resulting in the exclusion of 548 records. The remaining 269 full-text articles were assessed for eligibility, leading to the exclusion of 84 studies for the following reasons: not specific to type 2 diabetes (n=1), not SCI-indexed (n=4), not an NMA (n=13), duplicate publication (n=4), abstract only (n=48), ineligible interventions (n=12), and lack of quantitative analysis (n=2). Consequently, 185 studies were included for final data analysis and quality assessment. The list of excluded studies with specific reasons and the list of included studies are provided in the **Supplementary Material 3**.

### 3.2 Study characteristics

**Figure 2:**
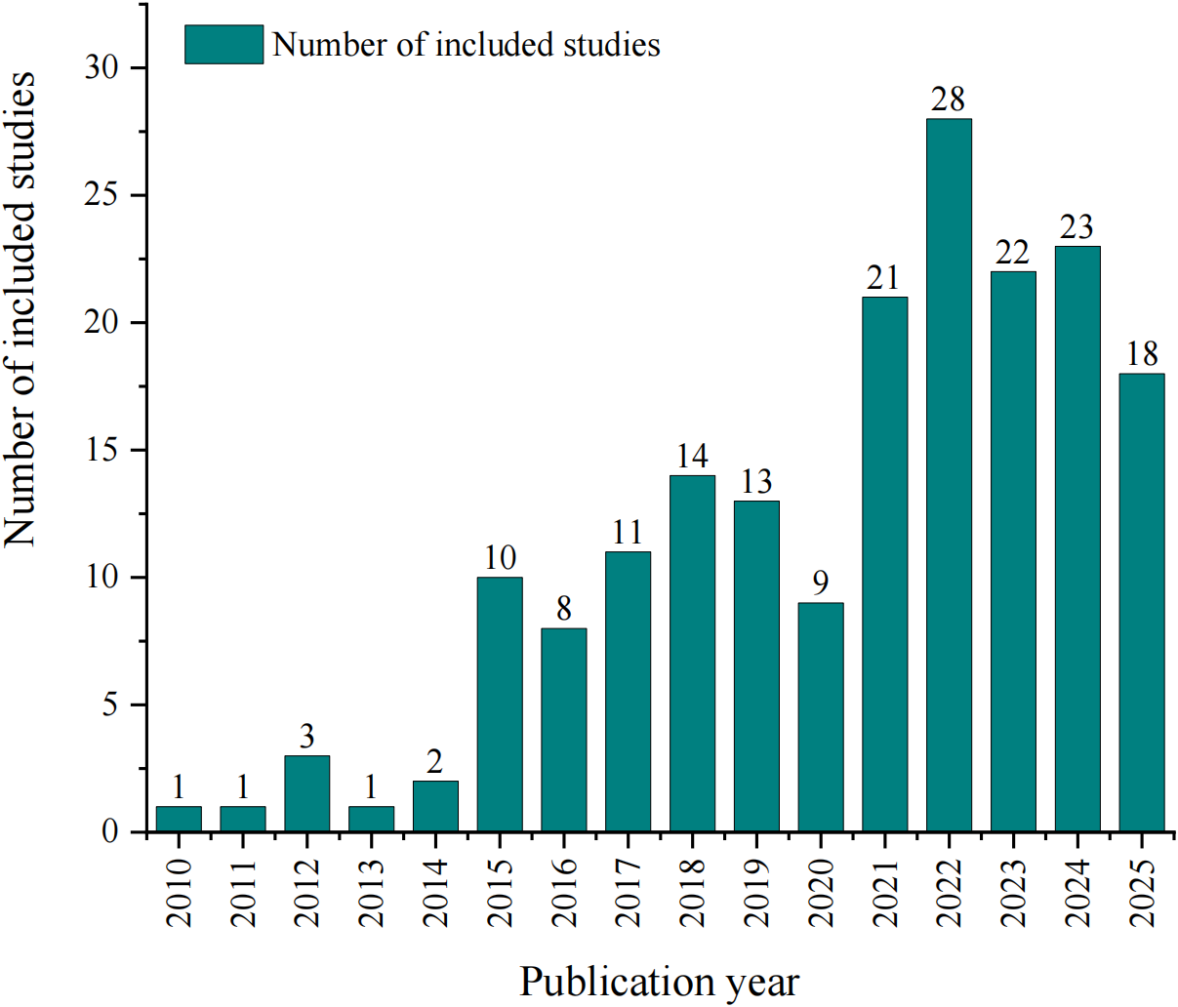
Number of included NMAs from 2010 to 2025.

The characteristics of the included studies are summarized in **Supplementary Material 4**. The annual number of published network meta-analyses (NMAs) demonstrated a general upward trend, with notable growth periods between 2014–2015 and 2020–2021, though a decline was observed after 2023. Publication frequency increased substantially following the release of AMSTAR 2 in 2017, with a statistically significant difference before and after the AMSTAR 2 publication (*p* = 0.02) (**Figure 2**). 108 studies (58.38%) originated from developing countries, compared to 77 (41.62%) from developed countries (*p* = 0.01). Asia contributed the majority of studies (122, 65.95%), with China accounting for 91 publications, while Africa had the fewest (1, 0.54%). Methodologically, most studies were published in high-impact journals, with 109 (58.92%) in JCR Q1 journals versus only 2 (1.08%) in Q4 journals (*p* = 0.03). The Bayesian approach was employed in 98 studies (52.97%), followed by frequentist methods (44, 23.78%), both methods (4, 2.16%), and unspecified methods (39, 21.08%), with significant differences among these categories (*p* < 0.001). The vast majority of NMAs (180, 97.30%) exclusively synthesized randomized controlled trials (RCTs), while 5 (2.70%) incorporated both RCTs and non-RCTs. The majority of studies involved comparisons of multiple antidiabetic drugs (122, 65.95%), while the remainder examined comparisons within the same drug class. Among these, studies on GLP-1 RAs were more commonly investigated (27, 14.59%), followed by SGLT2is (22, 11.89%), while DPP-4is (6, 3.24%) and Insulin (8, 4.32%) were the least studied. No statistically significant difference was found across the various intervention categories (*p* = 0.91). Nearly half of the included studies evaluated glycemic (88, 47.57%). A substantial number also focused on cardiovascular (33, 17.84%), kidney (9, 4.86%), and cardiovascular and kidney events (12, 6.49%). A smaller proportion examined safety outcomes (24, 12.97%), and other outcomes (neurological complications and gastrointestinal cancers) were also reported in a limited number of studies (19, 10.27%). No statistically significant difference was observed among the outcome categories (*p* = 0.24). More than three-quarters of the studies (142, 76.76%) reported funding sources, with a statistically significant difference between studies that reported funding sources and those that did not (*p* = 0.04). Over half of the studies (108, 58.38%) had pre-registration, and there was a statistically significant difference between protocol pre-registered and non-pre-registered (*p* < 0.001). The median impact factor was 4.60 (2.90–5.70). The median number of primary studies was 27.00 (14.00–54.00), and the median number of authors was 7.00 (5.00–10.00).

### 3.3 Reporting quality

A total of 185 NMAs were assessed and scored across the 32 items of the PRISMA-NMA statement. The median reporting quality score was 29.50 (IQR 27.50–30.50). Seventeen NMAs reported all checklist items, while the lowest score was 18.5. Based on the total scores, 67 NMAs (36.22%) were rated as high quality, 63 (34.05%) as moderate quality, and 55 (29.73%) as low quality. A statistically significant difference was observed among these reporting quality categories (*p* < 0.0001).

Regarding the compliance rate for each item, the items with 100% compliance were item 1 (“Title”) and item 26 (“Conclusion of Evidence”), indicating that all included literatures mentioned “network meta-analysis” in their titles, summarized the results in the discussion section, and clarified the implications for future research. Items with high compliance rates included: item 3 (“Rationale”, 99.73%), item 25 (“Limitations”, 99.73%), item 21 (“Results of NMA”, 99.19%), item 27 (“Funding”, 98.92%), item 24 (“Conclusions of main findings”, 98.92%), item 18 (“Characteristics of studies”, 98.92%), item 4 (“PICOS”, 98.92%), item 9 (“Study selection methods”, 98.38%), item 17 (“Results of study selection”, 98.11%), item 12 (“Risk of bias within studies”, 96.76%), item 14 (“Analysis methods”, 96.76%), item 2 (“Abstract”, 96.22%), item 13 (“Summary measures”, 95.68%), item 7 (“Information sources”, 94.59%), item 10 (“Data collection process”, 94.05%), item 20 (“Results of individual studies”, 91.62%), and item 15 (“Risk of bias across studies”, 90.81%).

**Figure 3:**
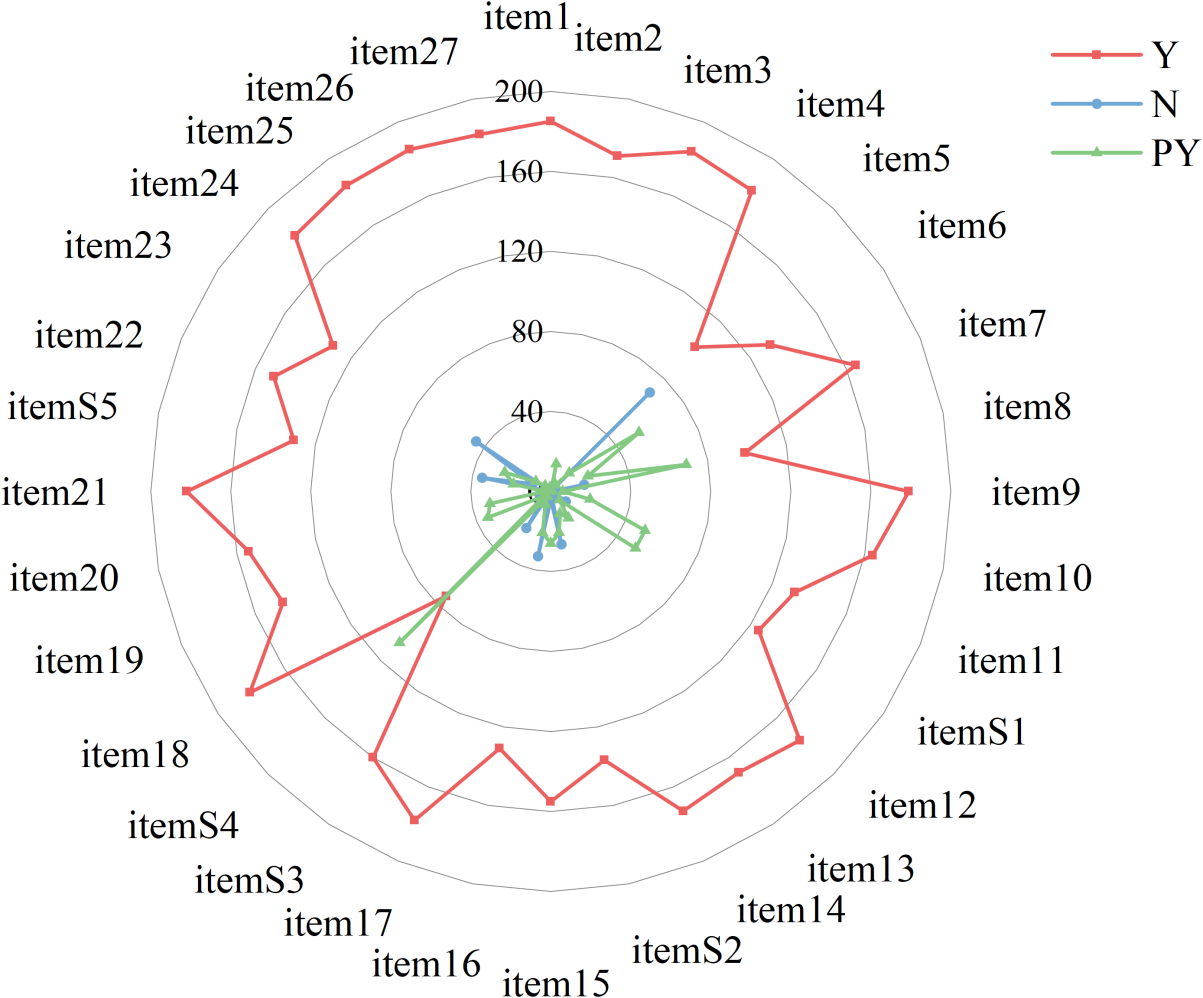
Reporting quality assessment of included NMAs. TITLE item 1: Title; ABSTRACT item 2: Structured summary; INTRODUCTION item 3: Rationale; item 4: Objectives; METHODS item 5: Protocol and registration; item 6: Eligibility criteria; item 7: Information sources; item 8: Search; item 9: Study selection; item 10: Data collection process; item 11: Data items; item S1: Geometry of the network; item 12: Risk of bias within individual studies; item 13: Summary measures; item 14: Planned methods of analysis; item S2: Assessment of inconsistency; item 15: Risk of bias across studies; item 16: Additional analyses; RESULTS item 17: Study selection; item S3: Presentation of network structure; item S4: Summary of network geometry; item 18: Study characteristics; item 19: Risk of bias within networks. Item 20: Results of individual studies; item 21: Synthesis of results; item S5: Exploration for consistency; item 22: Risk of bias across studies; item 23: Results of additional analyses; DISCUSSION item 24: Summary of evidence; item 25: Limitation; item 26: Conclusions; FUNDING item 27: Funding.

**Figure 4:**
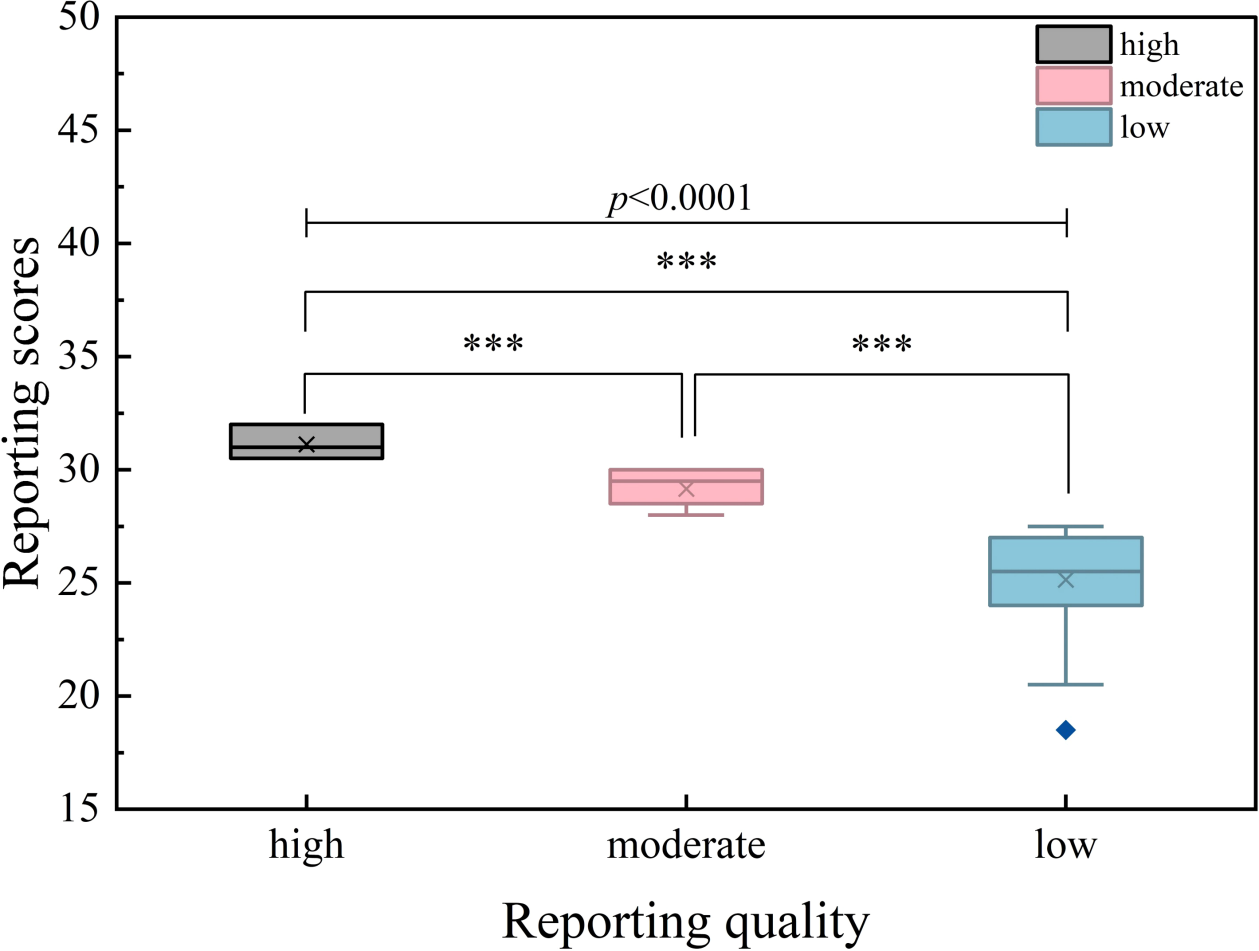
Box plot of reporting quality. ***: p<0.001.

In contrast, the reporting of methodology and results specific to network meta-analysis was notably poorer. Lower adherence was found for the following items: item S3 (“Presentation of network structure”, 85.68%), item S1 (“Geometry of the network”, 81.35%), item S2 (“Assessment of inconsistency”, 79.73%), item S4 (“Summary of network geometry”, 68.92%), and item S5 (“Exploration for inconsistency”, 58.65%). Specific details are shown on **Figure 3**, **Figure 4** and **Supplementary Material 5**.

### 3.4 Methodological quality

**Figure 5:**
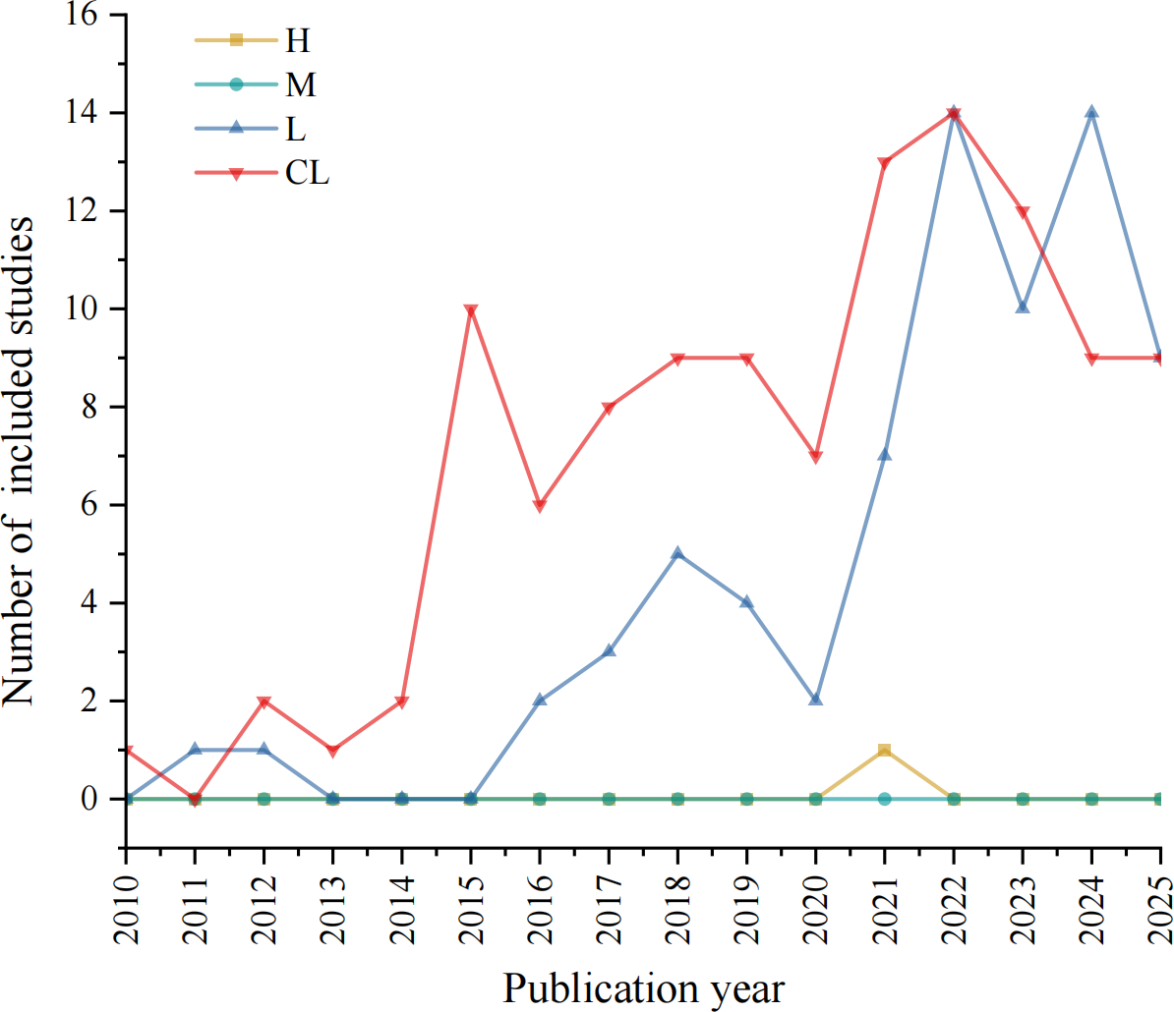
Methodological quality assessment of included NMAs per year. H, High; M, Moderate; L, Low; CL, Critically Low.

**Figure 6:**
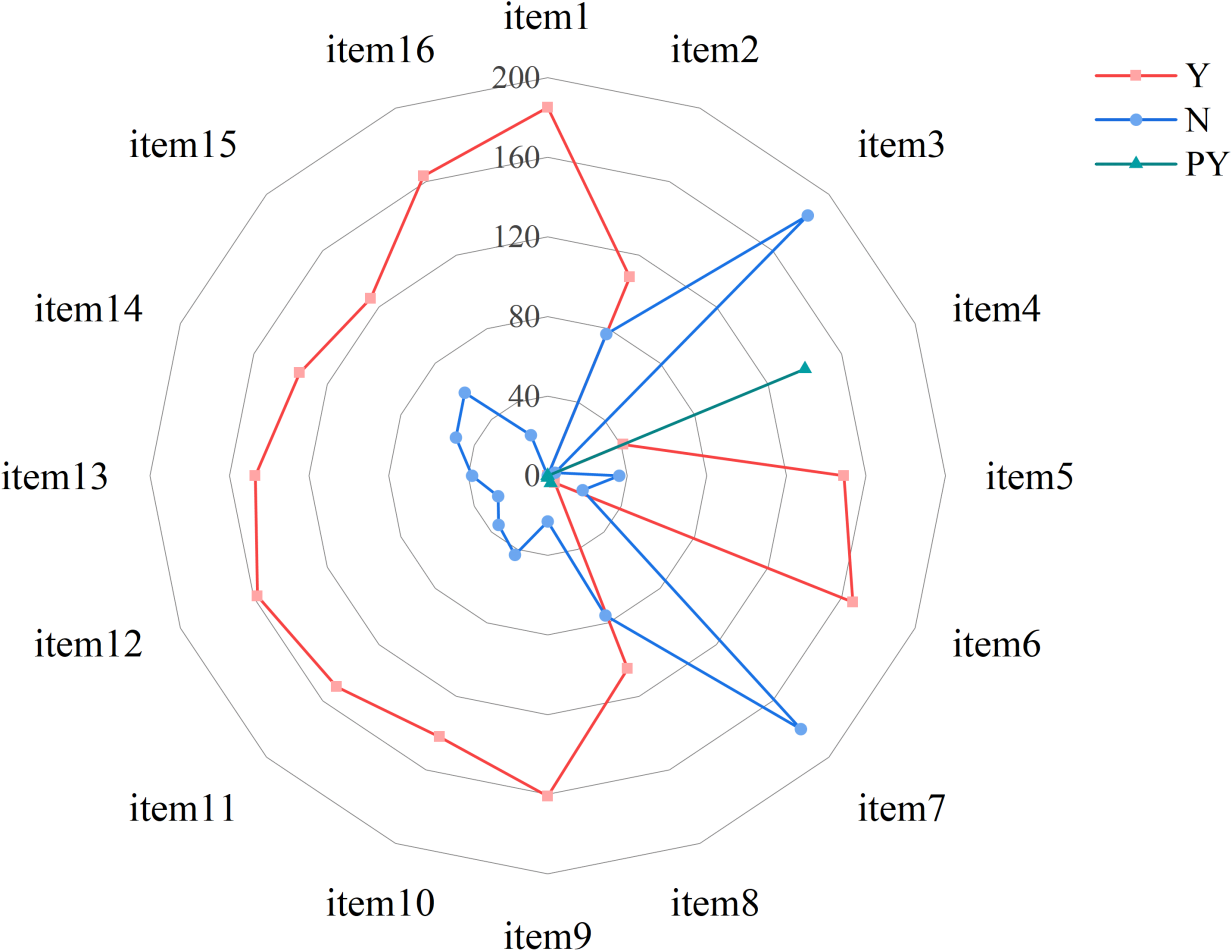
Methodological quality assessment of included NMAs. item 1: Did the research questions and inclusion criteria for the review include the components of PICO? item 2: Did the report of the review contain an explicit statement that the review methods were established prior to the conduct of the review and did the report justify any significant deviations from the protocol? item 3: Did the review authors explain their selection of the study designs for inclusion in the review? item 4: Did the review authors use a comprehensive literature search strategy? item 5: Did the review authors perform study selection in duplicate? item 6: Did the review authors perform data extraction in duplicate? item 7: Did the review authors provide a list of excluded studies and justify the exclusions? item 8: Did the review authors describe the included studies in adequate detail? item 9: Did the review authors use a satisfactory technique for assessing the risk of bias (RoB) in individual studies that were included in the review? item 10: Did the review authors report on the sources of funding for the studies included in the review? item 11: If meta-analysis was performed, did the review authors use appropriate methods for statistical combination of results? item 12: If meta-analysis was performed, did the review authors assess the potential impact of RoB in individual studies on the results of the meta-analysis or other evidence synthesis? item 13: Did the review authors account for RoB in primary studies when interpreting/discussing the results of the review? item 14: Did the review authors provide a satisfactory explanation for, and discussion of, any heterogeneity observed in the results of the review? item 15: If they performed quantitative synthesis, did the review authors carry out an adequate investigation of publication bias (small study bias) and discuss its likely impact on the results of the review? item 16: Did the review authors report any potential sources of conflict of interest, including any funding they received for conducting the review?

The methodological quality of the 185 included studies was rigorously assessed by the AMSTAR 2. Only 1 study (0.54%) was rated “High”, 72 studies (38.92%) “Low”, and 112 studies (60.54%) “Critically low”; no study was rated “Moderate”. Among the 184 NMAs that did not fully satisfy the critical items, 180 (97.83%) did not report item 7 (“Consideration of risk of bias when interpreting results”). More than half of the studies (76.09%) only partially report item 4 (“Adequacy of the literature search”). Additionally, less than half of the NMAs failed to report the following critical items: item 2 (“Protocol registered before commencement”, 41.85%), item 9 (“Risk of bias from individual studies used appropriately”, 12.50%), item 11 (“Appropriate methods for statistical combination”, 19.02%), item 13 (“Consideration of risk of bias when interpreting results”, 20.65%), and item 15 (“Assessment of presence and likely impact of publication bias”, 32.07%). Compliance with individual AMSTAR 2 items varied substantially. Item 1: “PICO” was fully reported by all studies (100%). Whereas item 3: “explanation of study design selection” was not reported by any study (0%). Similarly, Item 7 “list of excluded studies” was adequately addressed in only 2.70% of the studies. Several items were satisfactorily reported “Yes” in over 80% of the studies: Item 6: “two reviewers perform data extraction” (89.73%), Item 16: “conflict of interest” (88.11%), Item 9: “risk of bias” (87.03%), Item 12: “Impact of risk of bias” (85.41%), Item 11: “statistical methods” (81.08%), and Item 5: “two reviewers perform study selection” (80.54%). Reporting for Item 2 (protocol registration) was nearly evenly split between “Yes” (58.38%) and “No” (41.62%). Item 4: “study search strategy”, Item 8: “Detailed PICOS of the Included Studies”, and Item 9 were special, with the proportions of “partial Yes” reports being 75.68%, 41.08%, and 0.54% respectively. Specific details are shown on **Figure 5**, **Figure 6** and **Supplementary Material 6**.

Descriptive comparisons revealed significant heterogeneity in methodological quality in different subgroups. No statistically significant difference was observed among the different drug interventions (*p* = 0.59). “Comparisons of multiple antidiabetic drugs” constituted the highest proportion in both subgroups: specifically, in the critically low group (72, 64.29%) and the low group (49, 68.06%). Studies focusing on Insulin (8, 4.32%) and DPP-4is (6, 3.24%) accounted for a lower proportion in both groups. A statistically significant difference was observed across outcome categories (*p* = 0.04). The critically low group was predominantly comprised of studies focusing on glycemic (62, 55.36%), followed by cardiovascular (21, 18.75%). The low group also included a higher proportion of studies with glycemic (25, 34.72%), followed by safety outcomes (13, 18.06%). A statistically significant difference was noted among the analytical approaches. Bayesian methods were more frequently employed in the critically low group (71, 63.39%), whereas frequentist methods were more common in the low group (29, 40.28%). Approximately 20% of studies in both groups did not explicitly specify their analytical framework. These subgroup patterns should be interpreted as descriptive signals rather than inferential comparisons (**Supplementary Material 7**).

**Figure 7:**
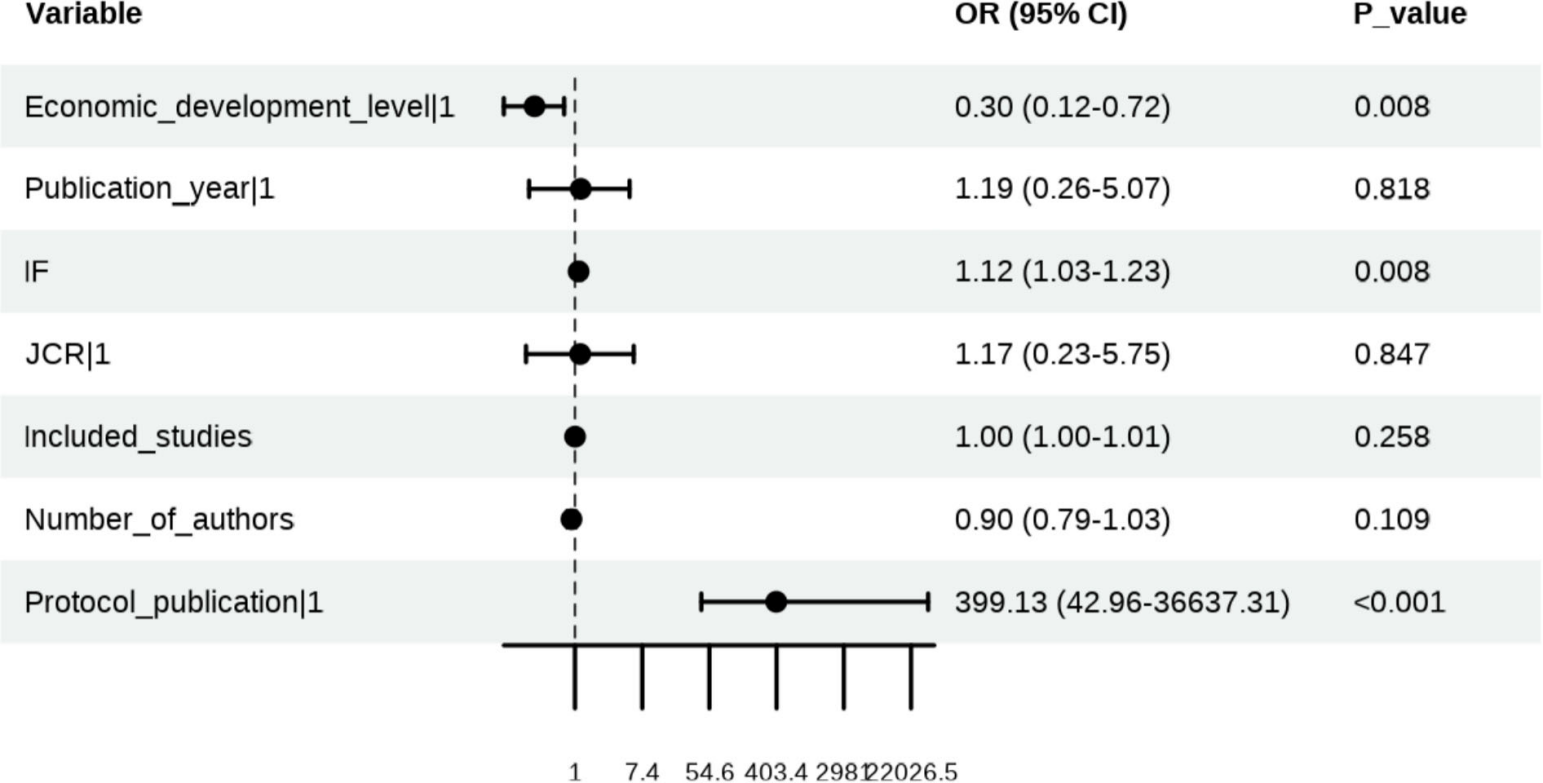
Forest plot of the multivariable regression analysis.

Given the imbalanced distribution of quality ratings, which could compromise model stability, the dependent variable was dichotomized for regression analysis. In the univariate analysis, several factors were significantly associated with higher methodological quality: studies published after the release of AMSTAR 2 (OR = 3.397, 95% CI = [1.39, 8.28], *p* = 0.007), higher journal impact factors (OR = 1.063, 95% CI = [1.01, 1.12], *p* = 0.017), a larger number of included studies (OR = 1.005, 95% CI = [1.00, 1.01], *p* = 0.012), protocol publication (compared with non-registration) (OR = 149.889, 95% CI = [19.75, 1137.43], *p* < 0.001), and higher JCR categories (OR = 4.636, 95% CI = [1.52, 14.10], *p* = 0.007). Conversely, studies from developed countries demonstrated lower quality (OR = 0.385, 95% CI = [0.20, 0.73], *p* = 0.003). No significant association was observed between the number of authors and study quality (OR = 1.063, 95% CI = [0.99, 1.14], *p* = 0.088). In the multivariate analysis adjusted for potential confounders, only three factors maintained statistical significance: protocol publication (OR = 399.130, 95% CI = [17.92, 8891.69], *p* < 0.001), and economic development level (OR = 0.380, 95% CI = [0.20, 0.73], *p* = 0.003). The following factors were no longer significantly associated with study quality in the adjusted model: journal impact factors (*p* = 0.008), publication year (*p* = 0.818), JCR category (*p* = 0.847), number of included studies (*p* = 0.258), and number of authors (*p* = 0.110). Specific details are shown on **Table 2**, **Table 3**, and **Figure 7**.

**Table 2:** Univariable regression analyses of methodological quality and independent variables.

Variable	OR	95%CI.low	95%CI.upp	*P*
Economic development level	0.385	0.20	0.73	0.003
Publication year	3.397	1.39	8.28	0.007
IF	1.063	1.01	1.12	0.017
JCR	4.636	1.52	14.10	0.007
Included studies	1.005	1.00	1.01	0.012
Number of authors	1.063	0.99	1.14	0.088
Protocol publication	149.889	19.75	1137.43	< 0.001

**Table 3:** Multiple regression analysis of methodological quality and independent variables.

Variable	OR	95%CI.low	95%CI.upp	*P*
Economic development level	0.301	0.12	0.74	0.008
Publication year	1.187	0.27	5.15	0.818
IF	1.116	1.03	1.21	0.008
JCR	1.167	0.24	5.63	0.847
Included studies	1.003	1.00	1.01	0.258
Number of authors	0.901	0.79	1.02	0.110
Protocol publication	399.128	17.92	8891.69	< 0.001

The multivariable regression analysis revealed an extremely elevated odds ratio for the variable “protocol registration status” (OR = 399.13). Statistical diagnostics indicated that this phenomenon was not caused by multicollinearity. Upon further investigation of the data characteristics, we found that among the included studies, those with a registered protocol were almost exclusively of higher quality. This distribution led to the extreme odds ratio and a wide confidence interval (17.92 – 8891.69). Nonetheless, the association remained statistically significant (*p* < 0.001), suggesting a strong correlation between protocol registration status and the methodological quality of the literature.

## 4 Discussion

### 4.1 Summary of findings

This study represents the first comprehensive assessment of the methodological quality of network meta-analyses (NMAs) that compares the efficacy of antidiabetic drugs for T2DM. Our analysis of 185 SCI-indexed NMAs revealed several key findings. Geographically, the majority of included studies originated from Asia and developing countries. While the annual number of NMAs on T2DM pharmacological treatment has shown a general increasing trend. Methodologically, over half of the NMAs employed Bayesian approaches for data synthesis. The majority of studies focused on Comparison of multiple antidiabetic drugs. Among studies comparing drugs within the same class, head-to-head comparisons of GLP-1 RAs were more commonly selected, while comparisons of DPP-4is were less frequent. Most studies primarily assessed glycemic, followed by cardiovascular and safety outcomes. Nearly all included studies were randomized controlled trials (RCTs), with very few non-randomized studies. However, none of the studies provided a rationale for their choice of study design, which may influence methodological quality. Overall, the general reporting quality of network meta-analyses was satisfactory, but notable deficiencies were observed in the reporting of methodology and results specific to NMA, primarily in the form of omissions or partial reporting. The methodological quality of most NMAs was rated low, with significant shortcomings in reporting critical items. Almost no studies reported on critical item 7, and only about half adequately addressed critical item 2. Therefore, researchers are advised to adopt more standardized practices in both reporting and methodological study design.

### 4.2 Implications of methodological flaws for clinical practice and future research

This study identified several methodological shortcomings in the network meta-analyses (NMAs) that compares the efficacy of antidiabetic drugs for T2DM published in SCI-indexed journals. These include a lack of protocol registration, failure to justify the selection of study design, and omission of searches in grey literature. In the absence of a registered protocol (29, 34), researchers might selectively include studies or outcomes based on preliminary results that favor their hypotheses or engage in excessive data dredging to achieve statistically significant findings. This practice can increase the likelihood of false-positive results while concealing unfavorable outcomes, thereby distorting conclusions and potentially misleading clinical decision-making, in addition to wasting research resources. Failure to provide a list of excluded studies with justifications compromises the reproducibility and transparency of the research. More critically, it may introduce selection bias, whereby researchers deliberately exclude undesirable results, skewing the final conclusions in a favorable direction. Furthermore, most studies did not provide a rationale for exclusively including randomized controlled trials (RCTs). While RCTs offer distinct advantages for evaluating drug efficacy, observational studies are often better suited for detecting long-term or rare adverse events. The exclusion of observational studies may lead to an underestimation or omission of such long-term safety outcomes, which are often difficult to identify in RCTs. The omission of grey literature is another significant concern. Grey literature may contain unpublished secondary outcomes, subgroup analyses, adverse event data, or important null results (35). Its absence can lead to a systematic overestimation of treatment benefits, obscure publication bias, and result in the omission of critical safety information.

In summary, methodologically flawed NMAs may lead clinicians and guideline developers to overinterpret imprecise or erroneous comparative findings. Therefore, enhancing the methodological rigor and reporting standards of network meta-analyses is crucial to ensure that treatment comparisons are not only statistically valid but also clinically reliable.

### 4.3 Improvement strategies

Several key methodological limitations were identified in the current landscape of network meta-analyses (NMAs). First, the majority of NMAs received low methodological quality ratings, primarily due to incomplete reporting of critical items. Notably, the processes of literature search, study selection, and the provision of a list of excluded studies with justifications were frequently omitted. Similarly, there was a marked deficiency in adhering to reporting guidelines specific to NMA. These shortcomings directly compromise the reliability of the evidence and may introduce bias into clinical decisions and guideline recommendations. Second, outcome selection remains heavily focused on glycemic, with relatively insufficient attention paid to cardiovascular, kidney, safety outcomes and other outcomes such as quality of life. This narrow focus may result in drug recommendation evidence that fails to fully capture a treatment’s risk profile, thereby increasing uncertainty in guideline development. Furthermore, most studies did not provide a rationale for exclusively including randomized controlled trials (RCTs), a critical component of methodological quality assessment. This omission may limit the generalizability of evidence to real-world clinical settings. Finally, nearly half of the reviewed studies lacked a preregistered protocol, which can lead to wasteful duplication of research efforts, increase the risk of data dredging, and directly undermine the study’s credibility.

Based on these findings, several practical strategies may enhance the methodological rigor of future NMAs in T2DM. First, routine protocol preregistration should be strongly encouraged to improve transparency and reduce selective reporting. Second, authors should explicitly justify their study design inclusion criteria and publish a complete list of excluded studies alongside the reasons for their exclusion. Third, reports should more clearly document the transitivity assumption, the assessment of inconsistency, and how the risk of bias from primary studies is considered within the network. Finally, guideline developers and journal editors could promote the use of structured methodological checklists to encourage more consistent reporting standards in NMAs. Implementing these approaches would help elevate the methodological and reporting quality of NMAs, thereby better serving evidence-based clinical decision-making.

### 4.4 Results of regression analysis

In the univariate regression analysis, economic development level, journal impact factor, and protocol registration status demonstrated significant associations with methodological quality; These associations remained statistically significant after full adjustment for confounding factors in multivariate analysis. Notably, protocol registration exhibited a substantially stronger association with quality than other independent variables, and further analysis confirmed this relationship was independent of multicollinearity. This finding underscores the critical role of protocol registration in enhancing the methodological rigor and reporting quality of research. Publication year, JCR category, and number of included studies were associated with literature quality in the univariate regression analysis, but this association disappeared after full adjustment for confounding factors. This discrepancy may be due to the fact that in the univariate analysis, the association of publication year, JCR category, and number of included studies with quality essentially stemmed from high impact factors—rather than the independent variables themselves. Therefore, after adjusting for the confounding variable of impact factor, the correlation between these independent variables and literature quality vanished. The number of authors demonstrated no significant association with methodological quality in either univariate or multivariate analyses, suggesting that research team size does not substantially influence methodological quality in this domain.

Our analysis revealed a consistent annual increase in the number of network meta-analyses comparing the efficacy of antidiabetic drugs for type 2 diabetes mellitus. This trend likely reflects both growing public health concern regarding type 2 diabetes and the increasingly widespread adoption of network meta-analysis methodology. In addition, the number of studies from developing countries included in this study was greater than that from developed countries. Combined with the regression analysis result that a lower level of economic development may be associated with a higher study quality rating, this phenomenon may be influenced by factors such as regional variations in journal selection policies, editorial standards, preregistration practices, or literature inclusion criteria.

### 4.5 Strength and limitations

There are a large number of network meta-analysis studies on the efficacy comparison of antidiabetic drugs for type 2 diabetes, but no thorough evaluation of the methodological quality of these studies has been performed to date. This study represents the first comprehensive methodological assessment of network meta-analyses that compares the efficacy of antidiabetic drugs for type 2 diabetes mellitus. The investigation employed rigorous methodology with transparent procedures, yielding reliable findings that provide valuable reference for future NMAs in this field. Furthermore, our regression analysis identified specific factors influencing methodological quality, offering evidence-based guidance for quality improvement initiatives.

However, this study still has some limitations. First, the AMSTAR 2 tool utilized in our assessment was not specifically designed for NMA methodology, potentially limiting its ability to evaluate certain NMA-specific methodological characteristics. Consequently, our results may not fully capture the comprehensive methodology quality landscape of the included NMAs. Although multicollinearity diagnostics indicated no severe collinearity among variables, the presence of extremely large effect estimates with wide confidence intervals for certain predictors suggests potential bias or error. Second, in order to ensure the applicability of the research results, this study only included studies from SCI-indexed journals. This may lead to the omission of some studies with regional characteristics that have not been included in SCI journals, thus limiting the application range of the research conclusions to a certain extent. Finally, the scarcity of high-quality studies in our sample necessitates cautious interpretation when extrapolating conclusions to high-quality NMAs.

Future research should address these limitations by developing an NMA-specific methodological assessment tool to enhance evaluation precision. The inclusion of non-SCI journals and regionally representative studies would significantly strengthen the comprehensiveness of future assessments. Additionally, while we adjusted for common confounding factors, residual confounding remains possible; further investigation should explore additional determinants of methodological quality to inform more targeted improvement strategies.

## 5 Conclusion

The overall reporting quality of NMAs in type 2 diabetes mellitus is relatively high, however, deficiencies persist in adherence to specific NMA reporting guidelines. The methodological quality remains generally low, particularly concerning the selection and exclusion of articles. Therefore, it is necessary to improve the study design of network meta-analyses in type 2 diabetes mellitus and develop a dedicated methodological assessment tool for network meta-analyses to enhance the overall quality.

## Data Availability

The original contributions presented in the study are included in the article/**Supplementary Material**. Further inquiries can be directed to the corresponding authors.

## Supplementary material

Supplementary Material 1 Search strategies.

Supplementary Material 2 Details of AMSTAR 2 rating approaches.

Supplementary Material 3 List of included studies, list of excluded studies and reasons.

Supplementary Material 4 baseline characteristics of included studies.

Supplementary Material 5 Results of PRISMA-NMA.

Supplementary Material 6 Results of AMSTAR2.
