# Supplementary material for: Methodological improvements are needed in network meta analyses of antidiabetic drugs for type 2 diabetes mellitus
Source: Front Endocrinol (Lausanne). 2026 Feb 25;17:1734108. doi: 10.3389/fendo.2026.1734108 (PMC12975571; doi:10.3389/fendo.2026.1734108)

**Supplementary material 1 Search strategies**

| **Databases [Platform]** Searches run July 2025 | **Results** |
| --- | --- |
| **PubMed** | 215 |
| **Cochrane Library** | 42 |
| **Embase** | 746 |
| **total** | 1003 |
| **Duplicate** | 186 |

**Database: PubMed <July 21st 2025> 215**

**Search Strategy:**

| **#** | **Searches** | **Results** |
| --- | --- | --- |
| #1 | (Diabetes Mellitus, Type 2[MeSH Terms]) OR ((((((((((((((((((((((((((((((((Diabetes Mellitus, Type 2[Title/Abstract]) OR (Diabetes Mellitus, Stable[Title/Abstract])) OR (Stable Diabetes Mellitus[Title/Abstract])) OR (Diabetes Mellitus, Noninsulin Dependent[Title/Abstract])) OR (Diabetes Mellitus, Adult-Onset[Title/Abstract])) OR (Adult-Onset Diabetes Mellitus[Title/Abstract])) OR (Diabetes Mellitus, Adult Onset[Title/Abstract])) OR (Diabetes Mellitus, Ketosis-Resistant[Title/Abstract])) OR (Diabetes Mellitus, Ketosis Resistant[Title/Abstract])) OR (Ketosis-Resistant Diabetes Mellitus[Title/Abstract])) OR (Diabetes Mellitus, Non-Insulin Dependent[Title/Abstract])) OR (Diabetes Mellitus, Non-Insulin-Dependent[Title/Abstract])) OR (Non-Insulin-Dependent Diabetes Mellitus[Title/Abstract])) OR (Diabetes Mellitus, Type II[Title/Abstract])) OR (NIDDM[Title/Abstract])) OR (Diabetes Mellitus, Maturity-Onset[Title/Abstract])) OR (Diabetes Mellitus, Maturity Onset[Title/Abstract])) OR (Maturity-Onset Diabetes Mellitus[Title/Abstract])) OR (Maturity Onset Diabetes Mellitus[Title/Abstract])) OR (MODY[Title/Abstract])) OR (Diabetes Mellitus, Slow-Onset[Title/Abstract])) OR (Diabetes Mellitus, Slow Onset[Title/Abstract])) OR (Slow-Onset Diabetes Mellitus[Title/Abstract])) OR (Type 2 Diabetes Mellitus[Title/Abstract])) OR (Noninsulin-Dependent Diabetes Mellitus[Title/Abstract])) OR (Noninsulin Dependent Diabetes Mellitus[Title/Abstract])) OR (Maturity-Onset Diabetes[Title/Abstract])) OR (Diabetes, Maturity-Onset[Title/Abstract])) OR (Maturity Onset Diabetes[Title/Abstract])) OR (Type 2 Diabetes[Title/Abstract])) OR (Diabetes, Type 2[Title/Abstract])) OR (Diabetes Mellitus, Noninsulin-Dependent[Title/Abstract])) | 267,059 |
| #2 | (((((((((((((((((network meta-analysis[Mesh])) OR (network meta analyses[Title/Abstract])) OR (NMA[Title/Abstract])) OR (mixed treatment comparison meta-analysis[Title/Abstract])) OR (mixed treatment comparisons meta analyses[Title/Abstract])) OR (mixed treatment meta-analysis[Title/Abstract])) OR (mixed treatment meta analyses[Title/Abstract])) OR (mixed treatment comparisons[Title/Abstract])) OR (mixed treatment comparison[Title/Abstract])) OR (multiple treatment comparison meta-analysis[Title/Abstract])) OR (multiple treatment comparisons meta analyses[Title/Abstract])) OR (multiple treatments meta-analysis[Title/Abstract])) OR (multiple treatments meta analyses[Title/Abstract])) OR (multiple treatment meta-analysis[Title/Abstract])) OR (multiple treatment meta analyses[Title/Abstract])) OR (multiple treatment comparison[Title/Abstract])) OR (multiple treatment comparisons[Title/Abstract]) | 7365 |
| #3 | #1 AND #2 | 215 |


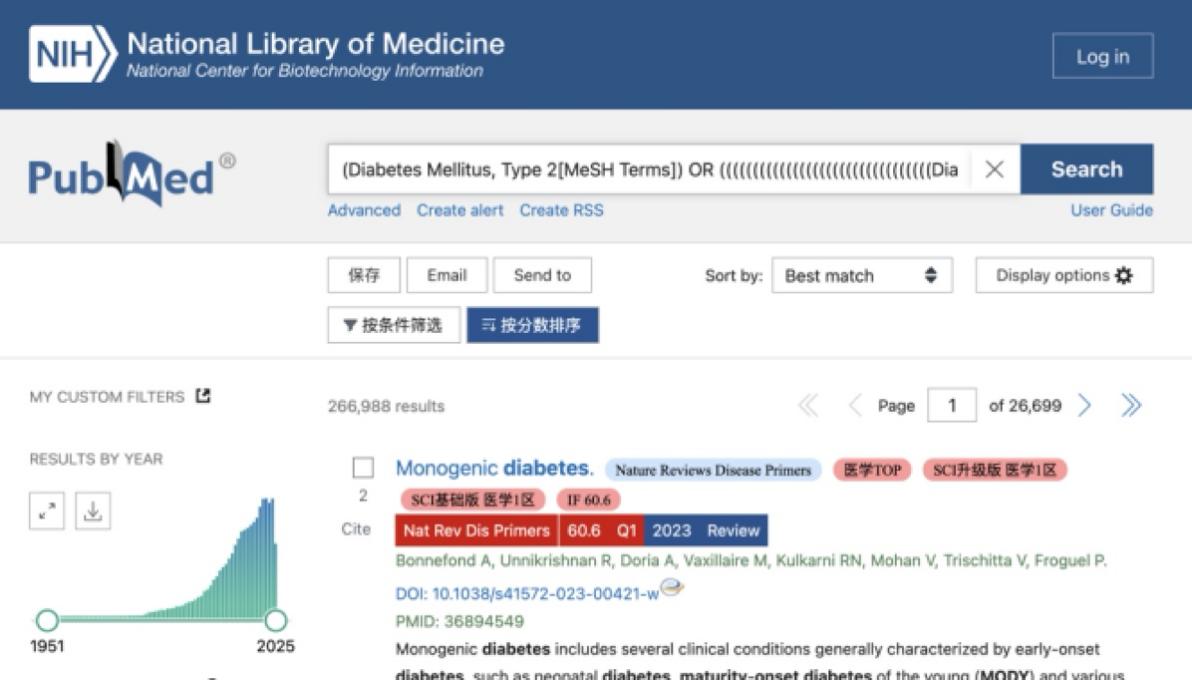

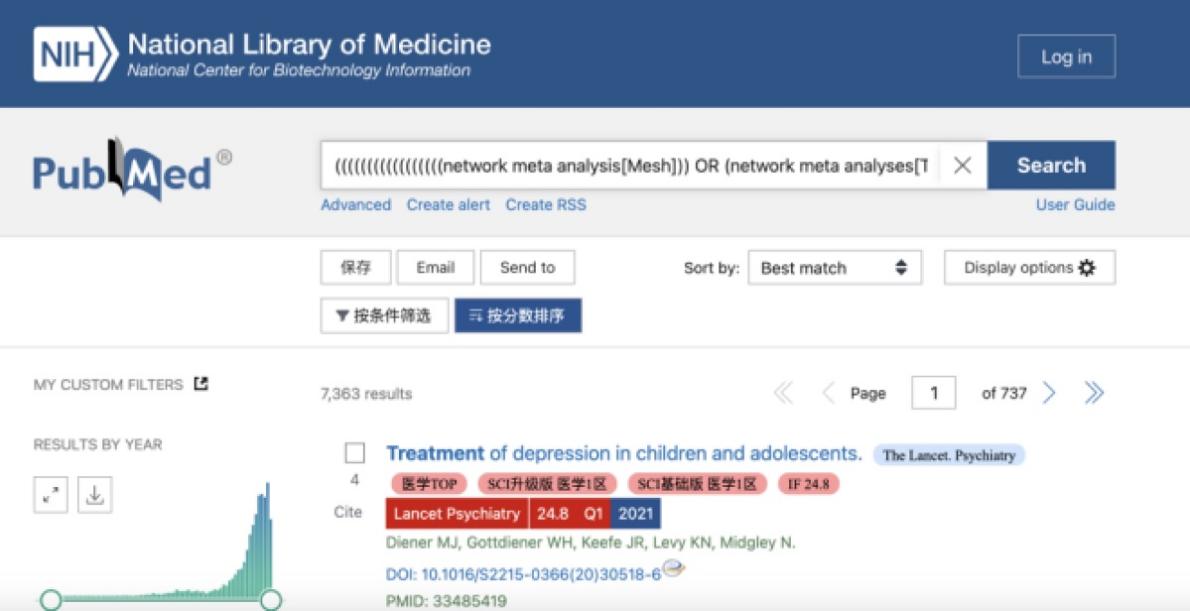


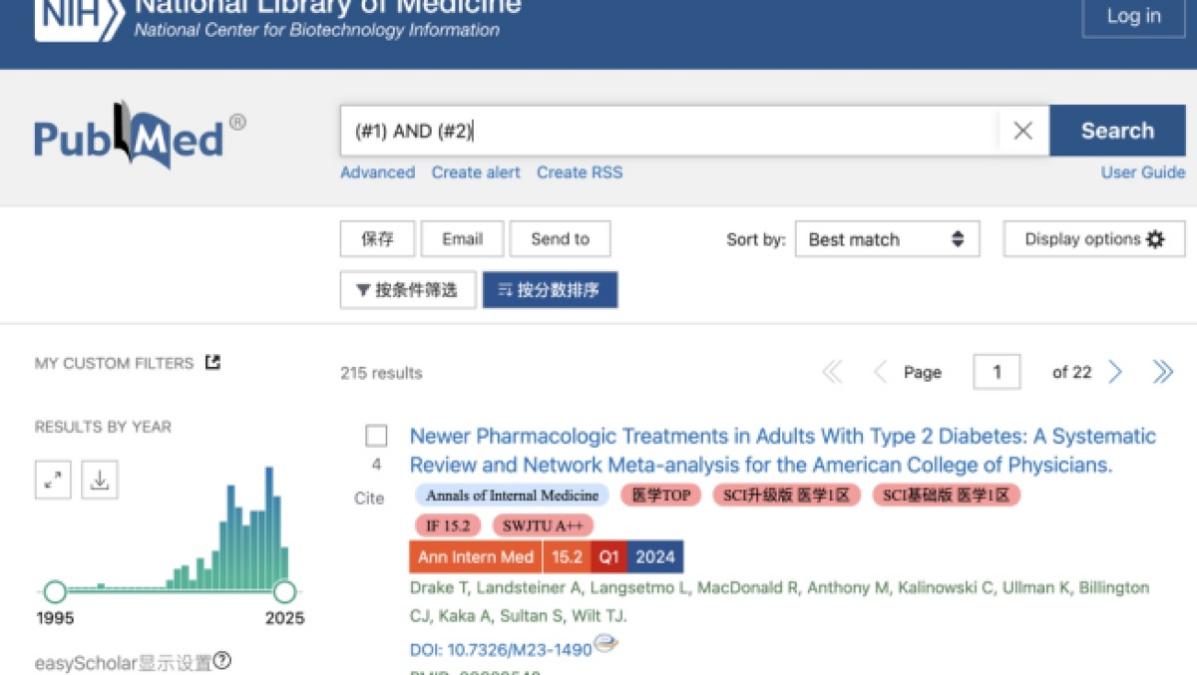


**Database: Cochrane Library <July 21st 2025> 42**

**Search Strategy:**

| **#** | **Searches** | **Results** |
| --- | --- | --- |
| #1 | MeSH descriptor: [Diabetes Mellitus, Type 2] explode all trees | 26788 |
| #2 | (Maturity-Onset Diabetes Mellitus):ti,ab,kw OR (Diabetes Mellitus, Slow-Onset):ti,ab,kw OR (Type 2 Diabetes):ti,ab,kw OR (Adult-Onset Diabetes Mellitus):ti,ab,kw OR (Ketosis-Resistant Diabetes Mellitus):ti,ab,kw OR (Diabetes Mellitus, Slow Onset):ti,ab,kw OR (Diabetes Mellitus, Noninsulin Dependent):ti,ab,kw OR (Non-Insulin-Dependent Diabetes Mellitus):ti,ab,kw OR (Diabetes Mellitus, Ketosis-Resistant):ti,ab,kw OR (Maturity-Onset Diabetes):ti,ab,kw OR (Diabetes Mellitus, Stable):ti,ab,kw OR (Diabetes Mellitus, Ketosis Resistant):ti,ab,kw OR (Diabetes Mellitus, Maturity Onset):ti,ab,kw OR (Maturity Onset Diabetes Mellitus):ti,ab,kw OR (Diabetes Mellitus, Adult Onset):ti,ab,kw OR (NIDDM):ti,ab,kw OR (Diabetes Mellitus, Adult-Onset):ti,ab,kw OR (Diabetes Mellitus, Maturity-Onset):ti,ab,kw OR (Noninsulin Dependent Diabetes Mellitus):ti,ab,kw OR (Diabetes, Type 2):ti,ab,kw OR (Diabetes, Maturity-Onset):ti,ab,kw OR (MODY):ti,ab,kw OR (Noninsulin-Dependent Diabetes Mellitus):ti,ab,kw OR (Diabetes Mellitus, Non-Insulin-Dependent):ti,ab,kw OR (Stable Diabetes Mellitus):ti,ab,kw | 68981 |
| #3 | #1 OR #2 | 68982 |
| #4 | ("network meta-analysis" OR "network meta analyses" OR "mixed treatment comparison meta-analysis" OR "mixed treatment comparisons meta analyses" OR "mixed treatment meta-analysis" OR "mixed treatment meta analyses" OR "mixed treatment comparisons" OR "mixed treatment comparison" OR "multiple treatment comparison meta-analysis" OR "multiple treatment comparisons meta analyses" OR "multiple treatments meta-analysis" OR "multiple treatments meta analyses" OR "multiple treatment meta-analysis" OR "multiple treatment meta analyses" OR "multiple treatment comparison" OR "multiple treatment comparisons"):ti,ab,kw | 875 |
| #5 | #3 AND #4 | 42 |


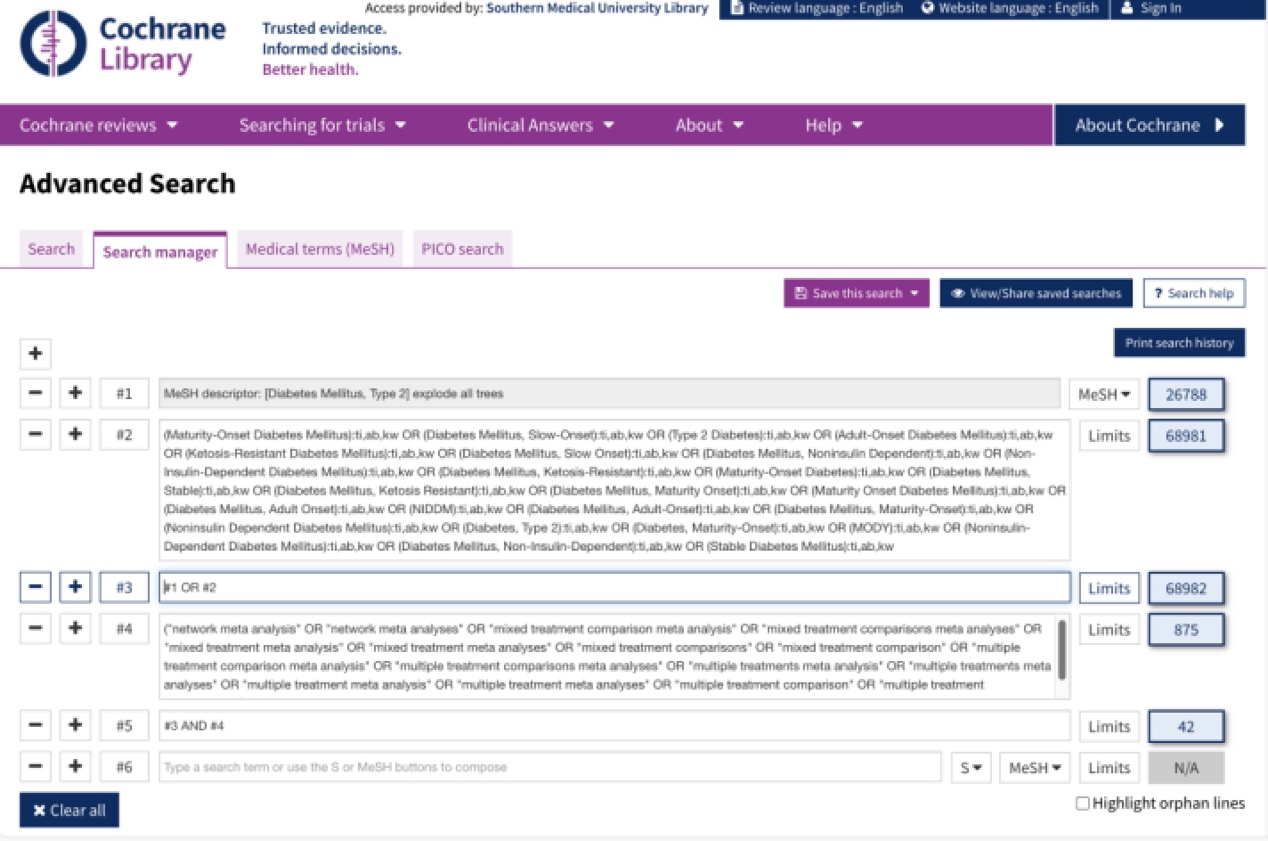


**Database: Embase <July 21st 2025> 746**

**Search Strategy:**

| **#** | **Searches** | **Results** |
| --- | --- | --- |
| #1 | ('non-insulin dependent diabetes mellitus'/exp OR 'non-insulin dependent diabetes mellitus') AND [embase]/lim | 369772 |
| #2 | (((('adult onset diabetes':ab,ti OR 'adult onset diabetes mellitus':ab,ti OR 'diabetes mellitus type 2':ab,ti OR 'diabetes mellitus type ii':ab,ti OR 'diabetes mellitus, maturity onset':ab,ti OR 'diabetes mellitus, non-insulin dependent':ab,ti OR 'diabetes mellitus, non-insulin-dependent':ab,ti OR 'diabetes mellitus, type 2':ab,ti OR 'diabetes mellitus, type ii':ab,ti OR 'diabetes type 2':ab,ti OR 'diabetes type ii':ab,ti OR 'diabetes, adult onset':ab,ti OR 'dm 2':ab,ti OR 'insulin independent diabetes':ab,ti OR 'insulin independent diabetes mellitus':ab,ti OR 'ketosis resistant diabetes mellitus':ab,ti OR 'maturity onset diabetes':ab,ti OR 'maturity onset diabetes mellitus':ab,ti OR 'niddm':ab,ti OR niddm:ab,ti) AND 'non-insulin dependent diabetes mellitus':ab,ti OR 'non-insulin dependent':ab,ti) AND 'type 2':ab,ti AND 'diabetes mellitus':ab,ti OR 'non-insulin dependent diabetes':ab,ti OR 'non-insulin-dependent diabetes mellitus':ab,ti OR 'noninsulin dependent':ab,ti) AND 'type 2':ab,ti AND 'diabetes mellitus':ab,ti OR 'noninsulin dependent diabetes':ab,ti OR 'noninsulin dependent diabetes mellitus':ab,ti OR 't2dm':ab,ti OR 'tiidm':ab,ti OR 'type 2':ab,ti) AND 'insulin independent':ab,ti AND diabetes:ab,ti OR 'type 2 diabetes':ab,ti OR 'type 2 diabetes mellitus':ab,ti OR 'type ii diabetes':ab,ti OR 'type ii diabetes mellitus':ab,ti OR 'non-insulin dependent diabetes mellitus':ab,ti | 325982 |
| #3 | #1 OR #2 | 458691 |
| #4 | 'network meta-analysis':ti,ab OR 'network meta analyses':ti,ab OR 'mixed treatment comparison meta-analysis':ti,ab OR 'mixed treatment comparisons meta analyses':ti,ab OR 'mixed treatment meta-analysis':ti,ab OR 'mixed treatment meta analyses':ti,ab OR 'mixed treatment comparisons':ti,ab OR 'mixed treatment comparison':ti,ab OR 'multiple treatment comparison meta-analysis':ti,ab OR 'multiple treatment comparisons meta analyses':ti,ab OR 'multiple treatments meta-analysis':ti,ab OR 'multiple treatments meta analyses':ti,ab OR 'multiple treatment meta-analysis':ti,ab OR 'multiple treatment meta analyses':ti,ab OR 'multiple treatment comparison':ti,ab OR 'multiple treatment comparisons':ti,ab | 16604 |
| #5 | #3 OR #4 | 746 |


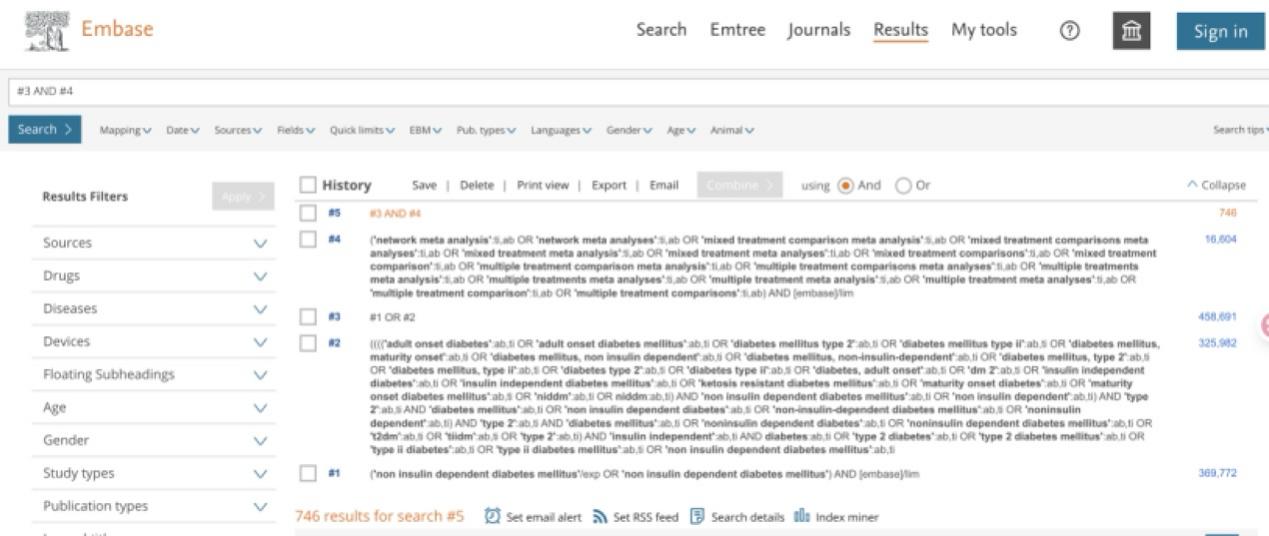

Supplement: Supplementary Material 1 — Search strategies. [file Supplementaryfile1.docx]
