# Supplementary material for: Methodological improvements are needed in network meta analyses of antidiabetic drugs for type 2 diabetes mellitus
Source: Front Endocrinol (Lausanne). 2026 Feb 25;17:1734108. doi: 10.3389/fendo.2026.1734108 (PMC12975571; doi:10.3389/fendo.2026.1734108)
Supplement: Supplementary Material 2 — Details of AMSTAR 2 rating approaches. [file Supplementaryfile2.docx]

**Supplementary material 2 Details of AMSTAR 2 rating approaches**

| High | No or one non-critical weakness: the systematic review provides an accurate and comprehensive summary of the results of the available studies that address the question of interest |
| --- | --- |
| Moderate | More than one non-critical weakness*: the systematic review has more than one weakness but no critical flaws. It may provide an accurate summary of the results of the available studies that were included in the review |
| Low | One critical flaw with or without non-critical weaknesses: the review has a critical flaw and may not provide an accurate and comprehensive summary of the available studies that address the question of interest |
| Critically low | More than one critical flaw with or without non-critical weaknesses: the review has more than one critical flaw and should not be relied on to provide an accurate and comprehensive summary of the available studies |

*Multiple non-critical weaknesses may diminish confidence in the review and it may be

appropriate to move the overall appraisal down from moderate to low confidence.
