# Supplementary material for: Methodological improvements are needed in network meta analyses of antidiabetic drugs for type 2 diabetes mellitus
Source: Front Endocrinol (Lausanne). 2026 Feb 25;17:1734108. doi: 10.3389/fendo.2026.1734108 (PMC12975571; doi:10.3389/fendo.2026.1734108)
Supplement: Supplementary Material 3 — List of included studies, list of excluded studies and reasons. [file Supplementaryfile3.docx]

**Supplementary material 3**

**List of included studies**

1. Kayaniyil S, Lozano-Ortega G, Bennett HA, Johnsson K, Shaunik A, Grandy S, et al. A Network Meta-analysis Comparing Exenatide Once Weekly with Other GLP-1 Receptor Agonists for the Treatment of Type 2 Diabetes Mellitus. Diabetes therapy. 2016;7:27–43. <https://doi.org/10.1007/s13300-016-0155-1>

2. Wang LG, Wang H, Liu Q, Hua WC, Li CM. A network meta-analysis for efficacy and safety of seven regimens in the treatment of type II diabetes. Biomedicine and Pharmacotherapy. 2017;92:707–19. <https://doi.org/10.1016/j.biopha.2017.05.002>

3. Scott DA, Boye KS, Timlin L, Clark JF, Best JH. A network meta-analysis to compare glycaemic control in patients with type 2 diabetes treated with exenatide once weekly or liraglutide once daily in comparison with insulin glargine, exenatide twice daily or placebo. Diabetes, Obesity and Metabolism. 2013;15:213–23. <https://doi.org/10.1111/dom.12007>

4. Witkowski M, Wilkinson L, Webb N, Weids A, Glah D, Vrazic H. A Systematic Literature Review and Network Meta-Analysis Comparing Once-Weekly Semaglutide with Other GLP-1 Receptor Agonists in Patients with Type 2 Diabetes Previously Receiving 1-2 Oral Anti-Diabetic Drugs. Diabetes Ther. 2018;9:1149–67. <https://doi.org/10.1007/s13300-018-0424-2>

5. Witkowski M, Wilkinson L, Webb N, Weids A, Glah D, Vrazic H. A Systematic Literature Review and Network Meta-Analysis Comparing Once-Weekly Semaglutide with Other GLP-1 Receptor Agonists in Patients with Type 2 Diabetes Previously Receiving Basal Insulin. Diabetes therapy. 2018;9:1233–51. <https://doi.org/10.1007/s13300-018-0428-y>

6. Zhang F, Xu S, Tang L, Pan X, Tong N. Acarbose with comparable glucose-lowering but superior weight-loss efficacy to dipeptidyl peptidase-4 inhibitors: A systematic review and network meta-analysis of randomized controlled trials. Frontiers in Endocrinology. 2020;11. <https://doi.org/10.3389/fendo.2020.00288>

7. Hanlon P, Butterly E, Wei L, Wightman H, Almazam SAM, Alsallumi K, et al. Age and Sex Differences in Efficacy of Treatments for Type 2 Diabetes: A Network Meta-Analysis. Jama. 2025;333:1062–73. <https://doi.org/10.1001/jama.2024.27402>

8. Li Z, Lin C, Cai X, Lv F, Yang W, Ji L. Anti-diabetic agents and the risks of dementia in patients with type 2 diabetes: a systematic review and network meta-analysis of observational studies and randomized controlled trials. Alzheimer’s Research and Therapy. 2024;16. <https://doi.org/10.1186/s13195-024-01645-y>

9. Tang H, Zhang B, Lu Y, Donahoo WT, Singh Ospina N, Kotecha P, et al. Assessing the benefit–risk profile of newer glucose-lowering drugs: A systematic review and network meta-analysis of randomized outcome trials. Diabetes, Obesity and Metabolism. 2025;27:1444–55. <https://doi.org/10.1111/dom.16147>

10. Zintzaras E, Miligkos M, Ziakas P, Balk EM, Mademtzoglou D, Doxani C, et al. Assessment of the relative effectiveness and tolerability of treatments of type 2 diabetes mellitus: A network meta-analysis. Clinical therapeutics. 2014;36:1443–53. <https://doi.org/10.1016/j.clinthera.2014.06.035>

11. Yang S, He W, Zhao L, Mi Y. Association between use of sodium-glucose cotransporter 2 inhibitors, glucagon-like peptide 1 agonists, and dipeptidyl peptidase 4 inhibitors with kidney outcomes in patients with type 2 diabetes: A systematic review and network meta-analysis. PLoS One. 2022;17. <https://doi.org/10.1371/journal.pone.0267025>

12. Wu T, Wong CKH, Lui DTW, Wong SKH, Lam CLK, Chung MSH, et al. Bariatric surgery, novel glucose-lowering agents, and insulin for type 2 diabetes and obesity: Bayesian network meta-analysis of randomized controlled trials. BJS Open. 2023;7. <https://doi.org/10.1093/bjsopen/zrad077>

13. Shi Q, Nong K, Vandvik PO, Guyatt GH, Schnell O, Rydén L, et al. Benefits and harms of drug treatment for type 2 diabetes: systematic review and network meta-analysis of randomised controlled trials. BMJ. 2023; <https://doi.org/10.1136/bmj-2022-074068>

14. Yan K, Yu H, Blaise B. Beyond GLP-1: efficacy and safety of dual and triple incretin agonists in personalized type 2 diabetes care—a systematic review and network meta-analysis. Acta Diabetologica. 2025; <https://doi.org/10.1007/s00592-025-02534-y>

15. Tsukamoto S, Morita R, Yamada T, Urate S, Azushima K, Uneda K, et al. Cardiovascular and kidney outcomes of combination therapy with sodium-glucose cotransporter-2 inhibitors and mineralocorticoid receptor antagonists in patients with type 2 diabetes and chronic kidney disease: A systematic review and network meta-analysis. Diabetes Research and Clinical Practice. 2022;194. <https://doi.org/10.1016/j.diabres.2022.110161>

16. Yamada T, Wakabayashi M, Bhalla A, Chopra N, Miyashita H, Mikami T, et al. Cardiovascular and renal outcomes with SGLT-2 inhibitors versus GLP-1 receptor agonists in patients with type 2 diabetes mellitus and chronic kidney disease: a systematic review and network meta-analysis. Cardiovascular Diabetology. 2021;20. <https://doi.org/10.1186/s12933-020-01197-z>

17. Tian L, Ai S, zheng H, Yang H, Zhou M, Tang J, et al. Cardiovascular and renal outcomes with sodium glucose co-transporter 2 inhibitors in patients with type 2 diabetes mellitus: A system review and network meta-analysis. Frontiers in pharmacology. 2022;13. <https://doi.org/10.3389/fphar.2022.986186>

18. Li J, Zhu C, Liang J, Hu J, Liu H, Wang Z, et al. Cardiovascular benefits and safety of sotagliflozin in type 2 diabetes mellitus patients with heart failure or cardiovascular risk factors: a bayesian network meta-analysis. Frontiers in pharmacology. 2023;14. <https://doi.org/10.3389/fphar.2023.1303694>

19. Sun F, Yu K, Wu S, Zhang Y, Yang Z, Shi L, et al. Cardiovascular safety and glycemic control of glucagon-like peptide-1 receptor agonists for type 2 diabetes mellitus: A pairwise and network meta-analysis. Diabetes Research and Clinical Practice. 2012;98:386–95. <https://doi.org/10.1016/j.diabres.2012.09.004>

20. Shi S, Li X, Chen Y, Li J, Dai Y. Cardiovascular Therapy Benefits of Novel Antidiabetic Drugs in Patients With Type 2 Diabetes Mellitus Complicated With Cardiovascular Disease: A Network Meta-Analysis. Journal of Diabetes. 2025;17. <https://doi.org/10.1111/1753-0407.70044>

21. Guo J, Wei M, Zhang W, Jiang Y, Li A, Wang C, et al. Clinical efficacy and safety of sodium-glucose cotransporter protein-2 (SGLT-2) inhibitor, glucagon-like peptide-1 (GLP-1) receptor agonist, and Finerenone in type 2 diabetes mellitus with non-dialysis chronic kidney disease: a network meta-analysis of randomized clinical trials. Frontiers in pharmacology. 2025;16:1517272. <https://doi.org/10.3389/fphar.2025.1517272>

22. Weng J, Tao Y, Xu Z, Zhou S, Xiao D, Zhu Z, et al. Comparation of fixed-ratio (IDegLira and iGlarLixi) versus free combination of basal insulin and glucagon-like peptide-1 receptor agonist for uncontrolled type 2 diabetes: A systematic review and network meta-analysis. Journal of Evidence-Based Medicine. 2024;17:370–6. <https://doi.org/10.1111/jebm.12620>

23. Madenidou AV, Paschos P, Karagiannis T, Katsoula A, Athanasiadou E, Kitsios K, et al. Comparative benefits and harms of basal insulin analogues for type 2 diabetes: A systematic review and network meta-analysis. Annals of internal medicine. 2018;169:165–74. <https://doi.org/10.7326/M18-0443>

24. Zhuo C, Lin C, Zhou C, Gao X, Shao H, Fang T, et al. Comparative Cardio-Renal Outcomes of Type 2 Diabetes Patients Administered Glucagon-Like Peptide-1 Receptor Agonists: A Network Meta-Analysis. Frontiers in pharmacology. 2021;12. <https://doi.org/10.3389/fphar.2021.759262>

25. Kongmalai T, Hadnorntun P, Leelahavarong P, Kongmalai P, Srinonprasert V, Chirakarnjanakorn S, et al. Comparative cardiovascular benefits of individual SGLT2 inhibitors in type 2 diabetes and heart failure: a systematic review and network meta-analysis of randomized controlled trials. Frontiers in Endocrinology. 2023;14. <https://doi.org/10.3389/fendo.2023.1216160>

26. Jiang Y, Yang P, Fu L, Sun L, Shen W, Wu Q. Comparative Cardiovascular Outcomes of SGLT2 Inhibitors in Type 2 Diabetes Mellitus: A Network Meta-Analysis of Randomized Controlled Trials. Frontiers in Endocrinology. 2022;13. <https://doi.org/10.3389/fendo.2022.802992>

27. Kim JS, Lee G, Park KI, Oh SW. Comparative Effect of Glucose-Lowering Drugs for Type 2 Diabetes Mellitus on Stroke Prevention: A Systematic Review and Network Meta-Analysis. Diabetes and Metabolism Journal. 2024;48:312–20. <https://doi.org/10.4093/dmj.2022.0421>

28. Kay S, Strickson A, Puelles J, Selby R, Benson E, Tolley K. Comparative Effectiveness of Adding Alogliptin to Metformin Plus Sulfonylurea with Other DPP-4 Inhibitors in Type 2 Diabetes: A Systematic Review and Network Meta-Analysis. Diabetes therapy. 2017;8:251–73. <https://doi.org/10.1007/s13300-017-0245-8>

29. Sim R, Chong CW, Loganadan NK, Fong AYY, Navaravong L, Hussein Z, et al. Comparative effectiveness of cardiovascular, renal and safety outcomes of second-line antidiabetic drugs use in people with type 2 diabetes: A systematic review and network meta-analysis of randomised controlled trials. Diabetic medicine. 2022;39. <https://doi.org/10.1111/dme.14780>

30. Yao H, Zhang A, Li D, Wu Y, Wang CZ, Wan JY, et al. Comparative effectiveness of GLP-1 receptor agonists on glycaemic control, body weight, and lipid profile for type 2 diabetes: Systematic review and network meta-analysis. BMJ. 2024; <https://doi.org/10.1136/bmj-2023-076410>

31. Tsapas A, Avgerinos I, Karagiannis T, Malandris K, Manolopoulos A, Andreadis P, et al. Comparative effectiveness of glucose-lowering drugs for type 2 diabetes: A systematic review and network meta-analysis. Annals of internal medicine. 2020;173:278–87. <https://doi.org/10.7326/M20-0864>

32. Orme ME, Nguyen H, Lu JY, Thomas SA. Comparative effectiveness of glycemic control in patients with type 2 diabetes treated with GLP-1 receptor agonists: A network meta-analysis of placebo-controlled and active-comparator trials. Diabetes, Metabolic Syndrome and Obesity. 2017;10:111–22. <https://doi.org/10.2147/DMSO.S116810>

33. Deng M, Wen Y, Yan JX, Fan Y, Wang Z, Zhang R, et al. Comparative effectiveness of multiple different treatment regimens for nonalcoholic fatty liver disease with type 2 diabetes mellitus: a systematic review and Bayesian network meta-analysis of randomised controlled trials. BMC Medicine. 2023;21. <https://doi.org/10.1186/s12916-023-03129-6>

34. Kim H, Park J, Gyu Kim M, Kim K. Comparative effects of antidiabetic drugs on arterial stiffness in type 2 diabetic patients: a systematic review and network meta-analysis. JACCP Journal of the American College of Clinical Pharmacy. 2022;5:1481. <https://doi.org/10.1002/jac5.1732>

35. Kim H, Choi CU, Rhew K, Park J, Lim Y, Kim MG, et al. Comparative effects of glucose-lowering agents on endothelial function and arterial stiffness in patients with type 2 diabetes: A network meta-analysis. Atherosclerosis. 2024;391. <https://doi.org/10.1016/j.atherosclerosis.2024.117490>

36. Zhang JJ, Huan Y, Leibensperger MR, Seo B, Song Y. Comparative effects of sodium-glucose cotransporter 2 inhibitors on serum electrolyte levels in patients with type 2 diabetes: A network meta-analysis of randomized controlled trials. Journal of the American Society of Nephrology. 2021;32:265. <https://doi.org/10.1681/asn.2020101466>

37. Xia L, Shen T, Dong W, Su F, Wang J, Wang Q, et al. Comparative efficacy and safety of 8 GLP-1RAs in patients with type 2 diabetes: A network meta-analysis. Diabetes Research and Clinical Practice. 2021;177. <https://doi.org/10.1016/j.diabres.2021.108904>

38. Mearns ES, Sobieraj DM, White CM, Saulsberry WJ, Kohn CG, Doleh Y, et al. Comparative efficacy and safety of antidiabetic drug regimens added to metformin monotherapy in patients with type 2 diabetes: A network meta-analysis. PLoS One. 2015;10. <https://doi.org/10.1371/journal.pone.0125879>

39. Zheng H, Sigal RJ, Coyle D, Bai Z, Johnston A, Elliott J, et al. Comparative efficacy and safety of antihyperglycemic drug classes for patients with type 2 diabetes following failure with metformin monotherapy: A systematic review and network meta-analysis of randomized controlled trials. Diabetes/Metabolism Research and Reviews. 2022;38. <https://doi.org/10.1002/dmrr.3515>

40. Wang G, Fu J, Li X, Wang J, Zhai J, Du B. Comparative efficacy and safety of dipeptidyl peptidase-4 inhibitors in adults with type 2 diabetes mellitus: A network meta-analysis. Diabetes, Obesity and Metabolism. 2025;27:1217–25. <https://doi.org/10.1111/dom.16114>

41. Chen H, Li XZ, Chen JQ, Ren TS, Zhang YS, Wang YN, et al. Comparative efficacy and safety of glucagon-like peptide 1 receptor agonists for the treatment of type 2 diabetes: A network meta-analysis. Medicine (United States). 2023;102:E34122. <https://doi.org/10.1097/MD.0000000000034122>

42. Wu S, He Y, Wu Y, Ji Y, Hou L, Liu X, et al. Comparative efficacy and safety of glucose-lowering drugs in children and adolescents with type 2 diabetes: A systematic review and network meta-analysis. Frontiers in Endocrinology. 2022;13. <https://doi.org/10.3389/fendo.2022.897776>

43. Su AY, Csere MM, Shan R, Pasupuleti V, Valenzuela GV, Hernandez AV. Comparative efficacy and safety of SGLT2 inhibitor class members in patients with heart failure and type 2 diabetes: A systematic review and network meta-analysis of randomized controlled trials. Diabetes Research and Clinical Practice. 2025;224. <https://doi.org/10.1016/j.diabres.2025.112219>

44. Ma J, Lu J, Shen P, Zhao X, Zhu H. Comparative efficacy and safety of sodium–glucose cotransporter 2 inhibitors for renal outcomes in patients with type 2 diabetes mellitus: a systematic review and network meta-analysis. Renal Failure. 2023;45. <https://doi.org/10.1080/0886022X.2023.2222847>

45. Ayesh H, Suhail S, Ayesh S, Niswender K. Comparative Efficacy and Safety of Weekly GLP-1/GIP Agonists vs. Weekly Insulin in Type 2 Diabetes: a Network Meta-Analysis of Randomized Controlled Trials. Biomedicines. 2024;12. <https://doi.org/10.3390/biomedicines12091943>

46. Ayesh H, Suhail S, Ayesh S, Niswender K. Comparative efficacy and safety of weekly tirzepatide versus weekly insulin in type 2 diabetes: A network meta-analysis of randomized clinical trials. Diabetes, Obesity and Metabolism. 2024;26:3801–9. <https://doi.org/10.1111/dom.15725>

47. Duan XY, Liu SY, Yin DG. Comparative efficacy of 5 sodium glucose cotransporter 2 inhibitor and 7 glucagon-like peptide 1 receptor agonists interventions on cardiorenal outcomes in type 2 diabetes patients: A network meta-analysis based on cardiovascular or renal outcome trials. Medicine (United States). 2021;100:E26431. <https://doi.org/10.1097/MD.0000000000026431>

48. Lautsch D, Alsumali A, McLeod E, Kuang Y, He J, Singh R, et al. Comparative Efficacy of Dual and Single Initiation of Add-On Oral Antihyperglycemic Agents in Type 2 Diabetes Uncontrolled on Metformin Alone: A Systematic Literature Review and Network Meta-Analysis. Diabetes therapy. 2021;12:389–418. <https://doi.org/10.1007/s13300-020-00975-y>

49. Tsapas A, Karagiannis T, Kakotrichi P, Avgerinos I, Mantsiou C, Tousinas G, et al. Comparative efficacy of glucose-lowering medications on body weight and blood pressure in patients with type 2 diabetes: A systematic review and network meta-analysis. Diabetes, Obesity and Metabolism. 2021;23:2116–24. <https://doi.org/10.1111/dom.14451>

50. Sharma R, Wilkinson L, Vrazic H, Popoff E, Lopes S, Kanters S, et al. Comparative efficacy of once-weekly semaglutide and SGLT-2 inhibitors in type 2 diabetic patients inadequately controlled with metformin monotherapy: a systematic literature review and network meta-analysis. Current medical research and opinion. 2018;34:1595–603. <https://doi.org/10.1080/03007995.2018.1476332>

51. Kanters S, Wilkinson L, Vrazic H, Sharma R, Lopes S, Popoff E, et al. Comparative efficacy of once-weekly semaglutide versus SGLT-2 inhibitors in patients inadequately controlled with one to two oral antidiabetic drugs: A systematic literature review and network meta-analysis. BMJ Open. 2019;9. <https://doi.org/10.1136/bmjopen-2018-023458>

52. Nguyen BN, Nguyen L, Mital S, Bugden S, Nguyen HV. Comparative efficacy of sodium-glucose co-transporter-2 inhibitors, glucagon-like peptide-1 receptor agonists and non-steroidal mineralocorticoid receptor antagonists in chronic kidney disease and type 2 diabetes: A systematic review and network meta-analysis. Diabetes, Obesity and Metabolism. 2023;25:1614–23. <https://doi.org/10.1111/dom.15009>

53. Täger T, Atar D, Agewall S, Katus HA, Grundtvig M, Cleland JGF, et al. Comparative efficacy of sodium-glucose cotransporter-2 inhibitors (SGLT2i) for cardiovascular outcomes in type 2 diabetes: a systematic review and network meta-analysis of randomised controlled trials. Heart Failure Reviews. 2021;26:1421–35. <https://doi.org/10.1007/s10741-020-09954-8>

54. Bae JH, Park EG, Kim S, Kim SG, Hahn S, Kim NH. Comparative renal effects of dipeptidyl peptidase-4 inhibitors and sodium-glucose cotransporter 2 inhibitors on individual outcomes in patients with type 2 diabetes: A systematic review and network meta-analysis. Endocrinology and Metabolism. 2021;36:388–400. <https://doi.org/10.3803/ENM.2020.912>

55. Chen L, Xue Q, Yan C, Tang B, Wang L, Zhang B, et al. Comparative safety of different recommended doses of sodium–glucose cotransporter 2 inhibitors in patients with type 2 diabetes mellitus: a systematic review and network meta-analysis of randomized clinical trials. Frontiers in Endocrinology. 2023;14. <https://doi.org/10.3389/fendo.2023.1256548>

56. Li CX, Liu LY, Zhang CX, Geng XH, Gu SM, Wang YQ, et al. Comparative safety of different sodium-glucose transporter 2 inhibitors in patients with type 2 diabetes: a systematic review and network meta-analysis of randomized controlled trials. Frontiers in Endocrinology. 2023;14. <https://doi.org/10.3389/fendo.2023.1238399>

57. Sabouret P, Bocchino PP, Angelini F, D’Ascenzo F, Galati G, Fysekidis M, et al. Comparing benefits from sodium-glucose cotransporter-2 inhibitors and glucagon-like peptide-1 receptor agonists in randomized clinical trials: a network meta-analysis. Minerva Cardiology and Angiology. 2023;71:199–207. <https://doi.org/10.23736/S2724-5683.22.05900-2>

58. Yuan X, Gao Z, Yang C, Duan K, Ren L, Song G. Comparing the effectiveness of long-term use of daily and weekly glucagon-like peptide-1 receptor agonists treatments in patients with nonalcoholic fatty liver disease and type 2 diabetes mellitus: a network meta-analysis. Frontiers in Endocrinology. 2023;14. <https://doi.org/10.3389/fendo.2023.1170881>

59. Qian D, Zhang T, Tan X, Zheng P, Liang Z, Xie J, et al. Comparison of antidiabetic drugs added to sulfonylurea monotherapy in patients with type 2 diabetes mellitus: A network meta-analysis. PLoS One. 2018;13. <https://doi.org/10.1371/journal.pone.0202563>

60. Visolyi GÁ, Domján BA, Svébis MM, Péterfi A, Lovász BD, Mészáros S, et al. Comparison of Efficacy and Safety of Commercially Available Fixed-Ratio Combinations of Insulin Degludec/Liraglutide and Insulin Glargine/Lixisenatide: A Network Meta-analysis. Canadian Journal of Diabetes. 2023;47:368–77. <https://doi.org/10.1016/j.jcjd.2023.03.002>

61. Li Y, Hu Y, Huyan X, Chen K, Li B, Gu W, et al. Comparison of efficacy and safety of three novel hypoglycemic agents in patients with severe diabetic kidney disease: A systematic review and network meta-analysis of randomized controlled trials. Frontiers in Endocrinology. 2022;13. <https://doi.org/10.3389/fendo.2022.1003263>

62. Hu J, Chen L. Comparison of glucagons like peptide-1 receptor agonists and dipeptidyl peptide-4 inhibitors regarding cardiovascular safety and mortality in type 2 diabetes mellitus: A network meta-analysis. Primary Care Diabetes. 2021;15:227–33. <https://doi.org/10.1016/j.pcd.2020.08.012>

63. Zaccardi F, Dhalwani NN, Dales J, Mani H, Khunti K, Davies MJ, et al. Comparison of glucose-lowering agents after dual therapy failure in type 2 diabetes: A systematic review and network meta-analysis of randomized controlled trials. Diabetes, Obesity and Metabolism. 2018;20:985–97. <https://doi.org/10.1111/dom.13185>

64. Boonpattharatthiti K, Wechkunanukul K, Mayang N, Lee EL, Fuangchan A, Cheng AYY, et al. Comparison of Insulin Titration Strategies for Glycemic Control in Type 2 Diabetes: A Systematic Review and Network Meta-analysis. Diabetes Care. 2025;48:837–45. <https://doi.org/10.2337/dc24-2661>

65. Men P, Qu S, Luo W, Li C, Zhai S. Comparison of lixisenatide in combination with basal insulin vs other insulin regimens for the treatment of patients with type 2 diabetes inadequately controlled by basal insulin: Systematic review, network meta-analysis and cost-effectiveness analysis. Diabetes, Obesity and Metabolism. 2020;22:107–15. <https://doi.org/10.1111/dom.13871>

66. Kramer CK, Ye C, Campbell S, Retnakaran R. Comparison of New Glucose-Lowering Drugs on Risk of Heart Failure in Type 2 Diabetes: A Network Meta-Analysis. JACC: Heart Failure. 2018;6:823–30. <https://doi.org/10.1016/j.jchf.2018.05.021>

67. Zhang X, Wang M, Wang X, Zhu Z, Zhang W, Zhou Z, et al. Comparison of New Glucose-Lowering Drugs on the Risk of Pancreatitis in Type 2 Diabetes: A Network Meta-Analysis. Endocrine Practice. 2022;28:333–41. <https://doi.org/10.1016/j.eprac.2021.12.007>

68. Wang M, Zhang X, Ni T, Wang Y, Wang X, Wu Y, et al. Comparison of New Oral Hypoglycemic Agents on Risk of Urinary Tract and Genital Infections in Type 2 Diabetes: A Network Meta-analysis. Advances in Therapy. 2021;38:2840–53. <https://doi.org/10.1007/s12325-021-01759-x>

69. Yoon JH, Min SH, Ahn CH, Cho YM, Hahn S. Comparison of non-insulin antidiabetic agents as an add-on drug to insulin therapy in type 2 diabetes: a network meta-analysis. Sci Rep. 2018;8:4095. <https://doi.org/10.1038/s41598-018-22443-1>

70. Qian D, Zhang T, Zheng P, Liang Z, Wang S, Xie J, et al. Comparison of Oral Antidiabetic Drugs as Add-On Treatments in Patients with Type 2 Diabetes Uncontrolled on Metformin: A Network Meta-Analysis. Diabetes therapy. 2018;9:1945–58. <https://doi.org/10.1007/s13300-018-0482-5>

71. Wen Z, Sun W, Wang H, Chang R, Wang J, Song C, et al. Comparison of the effectiveness and safety of GLP-1 receptor agonists for type 2 diabetes mellitus patients with overweight/obesity: A systematic review and network meta-analysis. Diabetes Research and Clinical Practice. 2025;222. <https://doi.org/10.1016/j.diabres.2025.111999>

72. Wei XB, Wei W, Ding LL, Liu SY. Comparison of the effects of 10 GLP-1 RA and SGLT2 inhibitor interventions on cardiovascular, mortality, and kidney outcomes in type 2 diabetes: A network meta-analysis of large randomized trials. Primary Care Diabetes. 2021;15:208–11. <https://doi.org/10.1016/j.pcd.2020.08.017>

73. Xie Z, Hu J, Gu H, Li M, Chen J. Comparison of the efficacy and safety of 10 glucagon-like peptide-1 receptor agonists as add-on to metformin in patients with type 2 diabetes: a systematic review. Frontiers in Endocrinology. 2023;14. <https://doi.org/10.3389/fendo.2023.1244432>

74. Tian S, Jiang J, Wang J, Zhang Z, Miao Y, Ji X, et al. Comparison on cognitive outcomes of antidiabetic agents for type 2 diabetes: A systematic review and network meta-analysis. Diabetes/Metabolism Research and Reviews. 2023;39. <https://doi.org/10.1002/dmrr.3673>

75. Li X, Wu H, Peng H, Jiang H. Comparison the effects of finerenone and SGLT2i on cardiovascular and renal outcomes in patients with type 2 diabetes mellitus: A network meta-analysis. Frontiers in Endocrinology. 2022;13. <https://doi.org/10.3389/fendo.2022.1078686>

76. Li H, Yang A, Zhao S, Chow EYK, Javanbakht M, Li Y, et al. Continuous Subcutaneous Insulin Infusion (CSII) Combined with Oral Glucose-Lowering Drugs in Type 2 Diabetes: A Systematic Review and Network Meta-Analysis of Randomized, Controlled Trials. Pharmaceuticals. 2022;15. <https://doi.org/10.3390/ph15080953>

77. He L, Wang J, Ping F, Yang N, Huang J, Li W, et al. Dipeptidyl peptidase-4 inhibitors and gallbladder or biliary disease in type 2 diabetes: systematic review and pairwise and network meta-analysis of randomised controlled trials. The BMJ. 2022; <https://doi.org/10.1136/bmj-2021-068882>

78. Hegde NC, Kumar A, Patil AN, Bhattacharjee S, Gamad N, Kasudhan KS, et al. Dose-dependent renoprotection efficacy of sglt2 inhibitors in type 2 diabetes: systematic review and network meta-analysis. Acta Diabetologica. 2023;60:1311–31. <https://doi.org/10.1007/s00592-023-02126-8>

79. Chen QQ, Yang Y, Xu JY, Wang J, Fang TY, Yuan Y, et al. Dose-Response Relationship of Glucagon-like Peptide-1 Receptor Agonists on HbA1c and Body Weight in Type 2 Diabetes Mellitus: A Systematic Review and Network Meta-Analysis. Endocrine Practice. 2025;31:188–97. <https://doi.org/10.1016/j.eprac.2024.11.013>

80. Liu SC, Tu YK, Chien MN, Chien KL. Effect of antidiabetic agents added to metformin on glycaemic control, hypoglycaemia and weight change in patients with type 2 diabetes: A network meta-analysis. Diabetes, Obesity and Metabolism. 2012;14:810–20. <https://doi.org/10.1111/j.1463-1326.2012.01606.x>

81. Gross JL, Kramer CK, Leitão CB, Hawkins N, Viana LV, Schaan BD, et al. Effect of antihyperglycemic agents added to metformin and a sulfonylurea on glycemic control and weight gain in type 2 diabetes: A network meta-analysis. Annals of internal medicine. 2011;154:672–9. <https://doi.org/10.7326/0003-4819-154-10-201105170-00007>

82. Sun F, Wu S, Guo S, Yu K, Yang Z, Li L, et al. Effect of GLP-1 receptor agonists on waist circumference among type 2 diabetes patients: a systematic review and network meta-analysis. Endocrine. 2015;48:794–803. <https://doi.org/10.1007/s12020-014-0373-0>

83. Sun F, Wu S, Wang J, Guo S, Chai S, Yang Z, et al. Effect of glucagon-like peptide-1 receptor agonists on lipid profiles among type 2 diabetes: A systematic review and network meta-analysis. Clinical therapeutics. 2015;37:225–41. <https://doi.org/10.1016/j.clinthera.2014.11.008>

84. Chai S, Yu S, Yang Z, Wu S, Gao L, Wang H, et al. Effect of incretin-based therapies on cancers of digestive system among 101 595 patients with type 2 diabetes mellitus: A systematic review and network meta-analysis combining 84 trials with a median duration of 30 weeks. BMJ Open Diabetes Research and Care. 2019;7. <https://doi.org/10.1136/bmjdrc-2019-000728>

85. Phung OJ, Scholle JM, Talwar M, Coleman CI. Effect of noninsulin antidiabetic drugs added to metformin therapy on glycemic control, weight gain, and hypoglycemia in type 2 diabetes. Jama. 2010;303:1410–8. <https://doi.org/10.1001/jama.2010.405>

86. Zhang YS, Zheng YD, Yuan Y, Chen SC, Xie BC. Effects of Anti-Diabetic Drugs on Fracture Risk: A Systematic Review and Network Meta-Analysis. Frontiers in Endocrinology. 2021;12. <https://doi.org/10.3389/fendo.2021.735824>

87. Wang Y, Yao M, Wang J, Liu H, Zhang X, Zhao L, et al. Effects of Antidiabetic Drugs on Endothelial Function in Patients With Type 2 Diabetes Mellitus: A Bayesian Network Meta-Analysis. Frontiers in Endocrinology. 2022;13. <https://doi.org/10.3389/fendo.2022.818537>

88. Guo Z, Huang L, Jiang Z, Bai X, Wang Z, Huang H. Effects of different hypoglycaemic drugs on beta-cell function in type 2 diabetes mellitus: a systematic review and network meta-analysis. European Journal of Medical Research. 2025;30. <https://doi.org/10.1186/s40001-025-02368-y>

89. Brønden A, Christensen MB, Glintborg D, Snorgaard O, Kofoed-Enevoldsen A, Madsen GK, et al. Effects of DPP-4 inhibitors, GLP-1 receptor agonists, SGLT-2 inhibitors and sulphonylureas on mortality, cardiovascular and renal outcomes in type 2 diabetes: A network meta-analyses-driven approach. Diabetic medicine. 2023;40. <https://doi.org/10.1111/dme.15157>

90. Sun F, Chai S, Li L, Yu K, Yang Z, Wu S, et al. Effects of glucagon-like peptide-1 receptor agonists on weight loss in patients with type 2 diabetes: A systematic review and network meta-analysis. Journal of Diabetes Research. 2015;2015. <https://doi.org/10.1155/2015/157201>

91. Xu L, Yu SQ, Gao L, Huang Y, Wu SS, Yang J, et al. Effects of Incretin-based Therapies on Weight-related Indicators among Patients with Type 2 Diabetes: A Network Meta-analysis. Biomed Environ Sci. 2020;33:37–47. <https://doi.org/10.3967/bes2020.005>

92. Ida S, Kaneko R, Imataka K, Okubo K, Shirakura Y, Azuma K, et al. Effects of oral antidiabetic drugs and glucagon-like peptide-1 receptor agonists on left ventricular diastolic function in patients with type 2 diabetes mellitus: a systematic review and network meta-analysis. Heart Failure Reviews. 2021;26:1151–8. <https://doi.org/10.1007/s10741-020-09936-w>

93. Ida S, Kaneko R, Murata K. Effects of oral antidiabetic drugs on left ventricular mass in patients with type 2 diabetes mellitus: A network meta-analysis. Cardiovascular Diabetology. 2018;17. <https://doi.org/10.1186/s12933-018-0773-1>

94. Wang H, Yang J, Chen X, Qiu F, Li J. Effects of Sodium-glucose Cotransporter 2 Inhibitor Monotherapy on Weight Changes in Patients With Type 2 Diabetes Mellitus: a Bayesian Network Meta-analysis. Clinical therapeutics. 2019;41:322-334.e11. <https://doi.org/10.1016/j.clinthera.2019.01.001>

95. Hu X, Yang Y, Hu X, Jia X, Liu H, Wei M, et al. Effects of sodium-glucose cotransporter 2 inhibitors on serum uric acid in patients with type 2 diabetes mellitus: A systematic review and network meta-analysis. Diabetes, Obesity and Metabolism. 2022;24:228–38. <https://doi.org/10.1111/dom.14570>

96. Morita R, Tsukamoto S, Obata S, Yamada T, Uneda K, Uehara T, et al. Effects of sodium-glucose cotransporter 2 inhibitors, mineralocorticoid receptor antagonists, and their combination on albuminuria in diabetic patients. Diabetes, Obesity and Metabolism. 2023;25:1271–9. <https://doi.org/10.1111/dom.14976>

97. Yang S, Zhao L, Mi Y, He W. Effects of sodium-glucose cotransporter-2 inhibitors and aldosterone antagonists, in addition to renin-angiotensin system antagonists, on major adverse kidney outcomes in patients with type 2 diabetes and chronic kidney disease: A systematic review and network meta-analysis. Diabetes, Obesity and Metabolism. 2022;24:2159–68. <https://doi.org/10.1111/dom.14801>

98. Zhong X, Zhang T, Liu Y, Wei X, Zhang X, Qin Y, et al. Effects of three injectable antidiabetic agents on glycaemic control, weight change and drop-out in type 2 diabetes suboptimally controlled with metformin and/or a sulfonylurea: A network meta-analysis. Diabetes Research and Clinical Practice. 2015;109:451–60. <https://doi.org/10.1016/j.diabres.2015.05.048>

99. Li Z, Zhang Y, Quan X, Yang Z, Zeng X, Ji L, et al. Efficacy and acceptability of glycemic control of glucagon-like peptide-1 receptor agonists among type 2 diabetes: A systematic review and network meta-analysis. PLoS One. 2016;11. <https://doi.org/10.1371/journal.pone.0154206>

100. Xu L, Wu Y, Li J, Ding Y, Chow J, Li L, et al. Efficacy and safety of 11 sodium-glucose cotransporter-2 inhibitors at different dosages in type 2 diabetes mellitus patients inadequately controlled with metformin: a Bayesian network meta-analysis. BMJ Open. 2025;15. <https://doi.org/10.1136/bmjopen-2024-088687>

101. Mearns ES, Saulsberry WJ, White CM, Kohn CG, Lemieux S, Sihabout A, et al. Efficacy and safety of antihyperglycaemic drug regimens added to metformin and sulphonylurea therapy in Type 2 diabetes: A network meta-analysis. Diabetic medicine. 2015;32:1530–40. <https://doi.org/10.1111/dme.12837>

102. Dehghani M, Sadeghi M, Barzkar F, Maghsoomi Z, Janani L, Motevalian SA, et al. Efficacy and safety of basal insulins in people with type 2 diabetes mellitus: a systematic review and network meta-analysis of randomized clinical trials. Frontiers in Endocrinology. 2024;15. <https://doi.org/10.3389/fendo.2024.1286827>

103. Mannucci E, Caiulo C, Naletto L, Madama G, Monami M. Efficacy and safety of different basal and prandial insulin analogues for the treatment of type 2 diabetes: a network meta-analysis of randomized controlled trials. Endocrine. 2021;74:508–17. <https://doi.org/10.1007/s12020-021-02889-6>

104. Chen R, Li J, Chen D, Wen W, Zhang S, Li J, et al. Efficacy and Safety of DPP-4 Inhibitors and Metformin Combinations in Type 2 Diabetes: A Systematic Literature Review and Network Meta-Analysis. Diabetes, Metabolic Syndrome and Obesity. 2024;17:2471–93. <https://doi.org/10.2147/DMSO.S450994>

105. Yang Q, Lang Y, Yang W, Yang F, Yang J, Wu Y, et al. Efficacy and safety of drugs for people with type 2 diabetes mellitus and chronic kidney disease on kidney and cardiovascular outcomes: A systematic review and network meta-analysis of randomized controlled trials. Diabetes Research and Clinical Practice. 2023;198. <https://doi.org/10.1016/j.diabres.2023.110592>

106. Wu Q, Liu M, Fang Z, Li C, Zou F, Hu L, et al. Efficacy and safety of empagliflozin at different doses in patients with type 2 diabetes mellitus: A network meta-analysis based on randomized controlled trials. Journal of Clinical Pharmacy and Therapeutics. 2022;47:270–86. <https://doi.org/10.1111/jcpt.13521>

107. Ren X, Hua H, Wu Y, Zhang W, Long X, Bai Y, et al. Efficacy and safety of GLP-1 agonists in the treatment of T2DM: A systematic review and network meta-analysis. Sci Rep. 2025;15:24103. <https://doi.org/10.1038/s41598-025-09807-0>

108. Jiang Y, Liu J, Chen X, Yang W, Jia W, Wu J. Efficacy and Safety of Glucagon-Like Peptide 1 Receptor Agonists for the Treatment of Type 2 Diabetes Mellitus: A Network Meta-analysis. Advances in Therapy. 2021;38:1470–82. <https://doi.org/10.1007/s12325-021-01637-6>

109. Mannucci E, Naletto L, Vaccaro G, Silverii A, Dicembrini I, Pintaudi B, et al. Efficacy and safety of glucose-lowering agents in patients with type 2 diabetes: A network meta-analysis of randomized, active comparator-controlled trials. Nutrition, Metabolism and Cardiovascular Diseases. 2021;31:1027–34. <https://doi.org/10.1016/j.numecd.2020.12.030>

110. Home P, Lauand F, Djaballah K, Li XT, Hafidh K, Mehta R, et al. Efficacy and safety of iGlarLixi versus IDegAsp in people with type 2 diabetes inadequately controlled with basal insulin: A systematic literature review and network meta-analysis of non-Asian studies. Diabetes, Obesity and Metabolism. 2025;27:3410–8. <https://doi.org/10.1111/dom.16360>

111. Nishimura R, Taniguchi M, Takeshima T, Iwasaki K. Efficacy and Safety of Metformin Versus the Other Oral Antidiabetic Drugs in Japanese Type 2 Diabetes Patients: A Network Meta-analysis. Advances in Therapy. 2022;39:632–54. <https://doi.org/10.1007/s12325-021-01979-1>

112. Wang P, Zhang Y, Xu W, He J, Peng L, Feng Y, et al. Efficacy and Safety of Once-Weekly Insulin Regimes on Glycemic Control for Type 2 Diabetes: A Systematic Review and Network Meta-analysis. Diabetology and Metabolic Syndrome. 2024;16. <https://doi.org/10.1186/s13098-023-01240-5>

113. Yang XL, Duo-Ji MM, Long ZW. Efficacy and Safety of Single- or Double-Drug Antidiabetic Regimens in the Treatment of Type 2 Diabetes Mellitus: A Network Meta-Analysis. Journal of Cellular Biochemistry. 2017;118:4536–47. <https://doi.org/10.1002/jcb.26115>

114. Zaccardi F, Webb DR, Htike ZZ, Youssef D, Khunti K, Davies MJ. Efficacy and safety of sodium-glucose co-transporter-2 inhibitors in type 2 diabetes mellitus: systematic review and network meta-analysis. Diabetes, Obesity and Metabolism. 2016;18:783–94. <https://doi.org/10.1111/dom.12670>

115. Shestakova MV, Galstyan GR, Kvasnikov BB, Erina EE. EFFICACY AND SAFETY OF SODIUM-GLUCOSE COTRANSPORTER-2 INHIBITORS IN TYPE 2 DIABETES MELLITUS WITH INADEQUATE GLYCEMIC CONTROL ON INSULIN: A NETWORK META-ANALYSIS. Diabetes Mellitus. 2024;27:543–54. <https://doi.org/10.14341/DM13244>

116. Zhu M, Guan R, Ma G. Efficacy and safety of teneligliptin in patients with type 2 diabetes mellitus: a Bayesian network meta-analysis. Frontiers in Endocrinology. 2023;14. <https://doi.org/10.3389/fendo.2023.1282584>

117. Osumili B, Sapin H, Yang Z, Ranta K, Paik JS, Blüher M. Efficacy and Safety of Tirzepatide Compared with GLP-1 RAs in Patients with Type 2 Diabetes Treated with Basal Insulin: A Network Meta-analysis. Diabetes therapy. 2025;16:1279–311. <https://doi.org/10.1007/s13300-025-01728-5>

118. Guan R, Yang Q, Yang X, Du W, Li X, Ma G. Efficacy and safety of tirzepatide in patients with type 2 diabetes mellitus: A bayesian network meta-analysis. Frontiers in pharmacology. 2022;13. <https://doi.org/10.3389/fphar.2022.998816>

119. Hussein H, Zaccardi F, Khunti K, Davies MJ, Patsko E, Dhalwani NN, et al. Efficacy and tolerability of sodium-glucose co-transporter-2 inhibitors and glucagon-like peptide-1 receptor agonists: A systematic review and network meta-analysis. Diabetes, Obesity and Metabolism. 2020;22:1035–46. <https://doi.org/10.1111/dom.14008>

120. Peng Y, Chen SH, Liu XN, Sun QY. Efficacy of different antidiabetic drugs based on metformin in the treatment of type 2 diabetes mellitus: A network meta-analysis involving eight eligible randomized-controlled trials. Journal of Cellular Physiology. 2019;234:2795–806. <https://doi.org/10.1002/jcp.27097>

121. McNeill AM, Davies G, Kruger E, Kowal S, Reason T, Ejzykowicz F, et al. Ertugliflozin Compared to Other Anti-hyperglycemic Agents as Monotherapy and Add-on Therapy in Type 2 Diabetes: A Systematic Literature Review and Network Meta-Analysis. Diabetes therapy. 2019;10:473–91. <https://doi.org/10.1007/s13300-019-0566-x>

122. Rangwala HS, Fatima H, Ali M, Mustafa MS, Shafique MA, Rangwala BS, et al. Evaluating the effectiveness and safety of various Tirzepatide dosages in the management of Type 2 diabetes mellitus: a network meta-analysis of randomized controlled trials. Journal of diabetes and metabolic disorders. 2024;23:1199‐1222. <https://doi.org/10.1007/s40200-024-01412-8>

123. Ding Y, Shi Y, Guan R, Yan S, Liu H, Wang Z, et al. Evaluation and comparison of efficacy and safety of tirzepatide and semaglutide in patients with type 2 diabetes mellitus: A Bayesian network meta-analysis. Pharmacological Research. 2024;199. <https://doi.org/10.1016/j.phrs.2023.107031>

124. Teng Y, Fan X, Yu R, Yang X. Evaluation and comparison of efficacy and safety of tirzepatide, liraglutide and SGLT2i in patients with type 2 diabetes mellitus: a network meta-analysis. BMC Endocrine Disorders. 2024;24. <https://doi.org/10.1186/s12902-024-01805-z>

125. Oh S, Park SE, Kim E. Evaluation of bone health and fracture risk in type 2 diabetes: a network meta-analysis of anti-diabetic treatments versus placebo. Archives of Pharmacal Research. 2025;48:563–75. <https://doi.org/10.1007/s12272-025-01552-2>

126. Pan SY, Su EL, Huang CJ, Chuang SY, Chiang CE, Chen CH, et al. Evaluation of glucose-lowering medications in older people: a comprehensive systematic review and network meta-analysis of randomized controlled trials. Age and Ageing. 2024;53. <https://doi.org/10.1093/ageing/afae175>

127. Gao H, Wei Q, Zou A, Yu K, Song D, Li J, et al. Evaluation of three mechanisms of action (SGLT2 inhibitors, GLP-1 receptor agonists, and sulfonylureas) in treating type 2 diabetes with heart failure: a systematic review and network meta-analysis of RCTs. Frontiers in Endocrinology. 2025;16. <https://doi.org/10.3389/fendo.2025.1562815>

128. Ghosal S, Sinha B. Exploring the comparative cardiovascular death benefits of sodium–glucose cotransporter 2 inhibitors in type 2 diabetes: a frequentist and Bayesian network meta-analysis-based scoring. Frontiers in Endocrinology. 2023;14. <https://doi.org/10.3389/fendo.2023.1168755>

129. Wu S, Chai S, Yang J, Cai T, Xu Y, Yang Z, et al. Gastrointestinal Adverse Events of Dipeptidyl Peptidase 4 Inhibitors in Type 2 Diabetes: A Systematic Review and Network Meta-analysis. Clinical therapeutics. 2017;39:1780-1789.e33. <https://doi.org/10.1016/j.clinthera.2017.07.036>

130. Sun F, Chai S, Yu K, Quan X, Yang Z, Wu S, et al. Gastrointestinal adverse events of glucagon-like peptide-1 receptor agonists in patients with type 2 diabetes: A systematic review and network meta-analysis. Diabetes Technology and Therapeutics. 2015;17:35–42. <https://doi.org/10.1089/dia.2014.0188>

131. Rayner CK, Wu T, Aroda VR, Whittington C, Kanters S, Guyot P, et al. Gastrointestinal adverse events with insulin glargine/lixisenatide fixed-ratio combination versus glucagon-like peptide-1 receptor agonists in people with type 2 diabetes mellitus: A network meta-analysis. Diabetes, Obesity and Metabolism. 2021;23:136–46. <https://doi.org/10.1111/dom.14202>

132. Zhang YS, Weng WY, Xie BC, Meng Y, Hao YH, Liang YM, et al. Glucagon-like peptide-1 receptor agonists and fracture risk: a network meta-analysis of randomized clinical trials. Osteoporosis international. 2018;29:2639–44. <https://doi.org/10.1007/s00198-018-4649-8>

133. Caruso I, Di Gioia L, Di Molfetta S, Cignarelli A, Palmer SC, Natale P, et al. Glucometabolic outcomes of GLP-1 receptor agonist-based therapies in patients with type 2 diabetes: a systematic review and network meta-analysis. eClinicalMedicine. 2023;64. <https://doi.org/10.1016/j.eclinm.2023.102181>

134. Grenet G, Ribault S, Nguyen GB, Glais F, Metge A, Linet T, et al. GLUcose COntrol Safety & Efficacy in type 2 DIabetes, a systematic review and NETwork meta-analysis. PLoS One. 2018;14. <https://doi.org/10.1371/journal.pone.0217701>

135. Grenet G, Ribault S, Nguyen GB, Glais F, Metge A, Linet T, et al. Glucose control safety & efficacy in type 2 diabetes, a systematic review and network meta-analysis (glucose dinet). European Journal of Clinical Pharmacology. 2019;75:S25. <https://doi.org/10.1007/s00228-019-02685-2>

136. Andersen SE, Christensen M. Hypoglycaemia when adding sulphonylurea to metformin: A systematic review and network meta-analysis. British Journal of Clinical Pharmacology. 2016; <https://doi.org/10.1111/bcp.13059>

137. Oh S, Purja S, Shin H, Kim M, Kim E. Hypoglycemic agents and glycemic variability in individuals with type 2 diabetes: A systematic review and network meta-analysis. Diabetes and Vascular Disease Research. 2022;19. <https://doi.org/10.1177/14791641221106866>

138. Yang Y, He L, Liu P, Wang J, Yang N, Li Z, et al. Impact of a dual glucose-dependent insulinotropic peptide/glucagon-like peptide-1 receptor agonist tirzepatide on heart rate among patients with type 2 diabetes: A systematic review and pairwise and network meta-analysis. Diabetes, Obesity and Metabolism. 2024;26:548–56. <https://doi.org/10.1111/dom.15342>

139. Sun F, Wu S, Guo S, Yu K, Yang Z, Li L, et al. Impact of GLP-1 receptor agonists on blood pressure, heart rate and hypertension among patients with type 2 diabetes: A systematic review and network meta-analysis. Diabetes Research and Clinical Practice. 2015;110:26–37. <https://doi.org/10.1016/j.diabres.2015.07.015>

140. Jia S, Wang Z, Han R, Zhang Z, Li Y, Qin X, et al. Incretin mimetics and sodium-glucose co-transporter 2 inhibitors as monotherapy or add-on to metformin for treatment of type 2 diabetes: a systematic review and network meta-analysis. Acta Diabetologica. 2021;58:5–18. <https://doi.org/10.1007/s00592-020-01542-4>

141. Alfayez OM, Almohammed OA, Alkhezi OS, Almutairi AR, Al Yami MS. Indirect comparison of glucagon like peptide-1 receptor agonists regarding cardiovascular safety and mortality in patients with type 2 diabetes mellitus: Network meta-analysis. Cardiovascular Diabetology. 2020;19. <https://doi.org/10.1186/s12933-020-01070-z>

142. Mitsuboshi S, Morizumi M, Kotake K, Kaseda R, Narita I. Individual dipeptidyl peptidase-4 inhibitors and acute kidney injury in patients with type 2 diabetes: A systematic review and network meta-analysis. Basic and Clinical Pharmacology and Toxicology. 2024;135:71–80. <https://doi.org/10.1111/bcpt.14014>

143. Home P, Blonde L, Kalra S, Ji L, Guyot P, Brulle-Wohlhueter C, et al. Insulin glargine/lixisenatide fixed-ratio combination (iGlarLixi) compared with premix or addition of meal-time insulin to basal insulin in people with type 2 diabetes: A systematic review and Bayesian network meta-analysis. Diabetes, Obesity and Metabolism. 2020;22:2179–88. <https://doi.org/10.1111/dom.14148>

144. Jia Y, Lao Y, Zhu H, Li N, Leung SW. Is metformin still the most efficacious first-line oral hypoglycaemic drug in treating type 2 diabetes? A network meta-analysis of randomized controlled trials. Obesity Reviews. 2019;20:1–12. <https://doi.org/10.1111/obr.12753>

145. Keshavarz K, Lotfi F, Sanati E, Salesi M, Hashemi-Meshkini A, Jafari M, et al. Linagliptin versus sitagliptin in patients with type 2 diabetes mellitus: A network meta-analysis of randomized clinical trials. DARU, Journal of Pharmaceutical Sciences. 2017;25. <https://doi.org/10.1186/s40199-017-0189-6>

146. Chae Y, Kwon SH, Nam JH, Kang E, Im J, Kim HJ, et al. Lipid profile changes induced by glucagon-like peptide-1 receptor agonists in patients with type 2 diabetes: a systematic review and network meta-analysis. Expert Review of Clinical Pharmacology. 2024;17:721–9. <https://doi.org/10.1080/17512433.2024.2363838>

147. Lorenzi M, Ploug UJ, Langer J, Skovgaard R, Zoratti M, Jansen J. Liraglutide Versus SGLT-2 Inhibitors in People with Type 2 Diabetes: A Network Meta-Analysis. Diabetes therapy. 2017;8:85–99. <https://doi.org/10.1007/s13300-016-0217-4>

148. Men P, Qu S, Song Z, Liu Y, Li C, Zhai S. Lixisenatide for Type 2 Diabetes Mellitus Patients Inadequately Controlled on Oral Antidiabetic Drugs: A Mixed-Treatment Comparison Meta-analysis and Cost-Utility Analysis. Diabetes Ther. 2020;11:1745–55. <https://doi.org/10.1007/s13300-020-00857-3>

149. Zilli RW, Rached CDA, Da Silva FP, Baena RC. Long-term efficacy of gliflozins versus gliptins for Type 2 Diabetes after metformin failure: A systematic review and network meta-analysis. Revista da Associacao Medica Brasileira. 2020;66:458–65. <https://doi.org/10.1590/1806-9282.66.4.458>

150. Simpson SH, Lee J, Choi S, Vandermeer B, Abdelmoneim AS, Featherstone TR. Mortality risk among sulfonylureas: A systematic review and network meta-analysis. The Lancet Diabetes and Endocrinology. 2015;3:43–51. <https://doi.org/10.1016/S2213-8587(14)70213-X>

151. Fei Y, Tsoi MF, Kumana CR, Cheung TT, Cheung BMY. Network meta-analysis of cardiovascular outcomes in randomised controlled trials of new antidiabetic drugs. Journal of the Hong Kong College of Cardiology. 2017;25:71.

152. Kodama S, Fujihara K, Ishiguro H, Matsubayashi Y, Kitazawa M, Iwanaga M, et al. Network meta-analysis of glucose-lowering drug treatment regimens with the potential risk of hypoglycemia in patients with type 2 diabetes mellitus in terms of glycemic control and severe hypoglycemia. Journal of Investigative Medicine. 2023;71:400–10. <https://doi.org/10.1177/10815589221149188>

153. Ayers D, Kanters S, Goldgrub R, Hughes M, Kato R, Kragh N. Network meta-analysis of liraglutide versus dipeptidyl peptidase-4 inhibitors for the treatment of type 2 diabetes in Japanese patients. Current medical research and opinion. 2017;33:1653–61. <https://doi.org/10.1080/03007995.2017.1345730>

154. Neff LM, Broder MS, Beenhouwer D, Chang E, Papoyan E, Wang ZW. Network meta-analysis of lorcaserin and oral hypoglycaemics for patients with type 2 diabetes mellitus and obesity. Clinical Obesity. 2017;7:337–46. <https://doi.org/10.1111/cob.12213>

155. Alfayez OM, Al Yami MS, Alshibani M, Fallatah SB, Al Khushaym NM, Alsheikh R, et al. Network meta-analysis of nine large cardiovascular outcome trials of new antidiabetic drugs. Primary Care Diabetes. 2019;13:204–11. <https://doi.org/10.1016/j.pcd.2019.01.003>

156. Zhang Y, Jiang L, Wang J, Wang T, Chien C, Huang W, et al. Network meta-analysis on the effects of finerenone versus SGLT2 inhibitors and GLP-1 receptor agonists on cardiovascular and renal outcomes in patients with type 2 diabetes mellitus and chronic kidney disease. Cardiovascular Diabetology. 2022;21. <https://doi.org/10.1186/s12933-022-01676-5>

157. Zhao LM, Zhan ZL, Ning J, Qiu M. Network Meta-Analysis on the Effects of SGLT2 Inhibitors Versus Finerenone on Cardiorenal Outcomes in Patients With Type 2 Diabetes and Chronic Kidney Disease. Frontiers in pharmacology. 2022;12. <https://doi.org/10.3389/fphar.2021.751496>

158. Zhang Y, Wang J, Jiang L, Wang T, Li Z, Fu X, et al. Network meta-analysis on the efficacy and safety of finerenone versus SGLT2 inhibitors on reducing new-onset of atrial fibrillation in patients with type 2 diabetes mellitus and chronic kidney disease. Diabetology and Metabolic Syndrome. 2022;14. <https://doi.org/10.1186/s13098-022-00929-3>

159. Gao L, Yu S, Cipriani A, Wu S, Huang Y, Zhang Z, et al. Neurological manifestation of incretin-based therapies in patients with type 2 diabetes: A systematic review and network meta-analysis. Aging and Disease. 2019;10:1311–9. <https://doi.org/10.14336/AD.2019.0303>

160. Drake T, Landsteiner A, Langsetmo L, MacDonald R, Anthony M, Kalinowski C, et al. Newer Pharmacologic Treatments in Adults With Type 2 Diabetes: A Systematic Review and Network Meta-analysis for the American College of Physicians. Annals of internal medicine. 2024;177. <https://doi.org/10.7326/M23-1490>

161. Chubb B, Gupta P, Gupta J, Nuhoho S, Kallenbach K, Orme M. Once-Daily Oral Semaglutide Versus Injectable GLP-1 RAs in People with Type 2 Diabetes Inadequately Controlled on Basal Insulin: Systematic Review and Network Meta-analysis. Diabetes therapy. 2021;12:1325–39. <https://doi.org/10.1007/s13300-021-01034-w>

162. Nuhoho S, Gupta J, Hansen BB, Fletcher-Louis M, Dang-Tan T, Paine A. Orally Administered Semaglutide Versus GLP-1 RAs in Patients with Type 2 Diabetes Previously Receiving 1-2 Oral Antidiabetics: Systematic Review and Network Meta-Analysis. Diabetes Ther. 2019;10:2183–99. <https://doi.org/10.1007/s13300-019-00706-y>

163. Lin J, Wang S, Wen T, Zhang X. Renal protective effect and safety of sodium-glucose cotransporter-2 inhibitors in patients with chronic kidney disease and type 2 diabetes mellitus: a network meta-analysis and systematic review. International Urology and Nephrology. 2022;54:2305–16. <https://doi.org/10.1007/s11255-022-03117-4>

164. Mostafa MEA, Alrasheed T. Risk of bone fracture by using dipeptidyl peptidase-4 inhibitors, glucagon-like peptide-1 receptor agonists, or sodium-glucose cotransporter-2 inhibitors in patients with type 2 diabetes mellitus: a network meta-analysis of population-based cohort studies. Frontiers in Endocrinology. 2024;15. <https://doi.org/10.3389/fendo.2024.1410883>

165. Tsai WH, Kong SK, Tsai MC. Risk of fracture caused by anti-diabetic drugs in individuals with type 2 diabetes: A network meta-analysis. Diabetes. 2022;71. <https://doi.org/10.2337/db22-181-LB>

166. Chai S, Liu F, Yang Z, Yu S, Liu Z, Yang Q, et al. Risk of Fracture With Dipeptidyl Peptidase-4 Inhibitors, Glucagon-like Peptide-1 Receptor Agonists, or Sodium-Glucose Cotransporter-2 Inhibitors in Patients With Type 2 Diabetes Mellitus: A Systematic Review and Network Meta-analysis Combining 177 Randomized Controlled Trials With a Median Follow-Up of 26 weeks. Frontiers in pharmacology. 2022;13. <https://doi.org/10.3389/fphar.2022.825417>

167. Tricco AC, Antony J, Khan PA, Ghassemi M, Hamid JS, Ashoor H, et al. Safety and effectiveness of dipeptidyl peptidase-4 inhibitors versus intermediate-acting insulin or placebo for patients with type 2 diabetes failing two oral antihyperglycaemic agents: A systematic review and network meta-analysis. BMJ Open. 2014;4. <https://doi.org/10.1136/bmjopen-2014-005752>

168. Freemantle N, Wang H, Thakur D, Stella P, Chanan N, Kalra M, et al. Safety and efficacy of insulin glargine 300 u/ml (GLA-300) compared with other basal or premixed insulin therapies in patients with type 2 diabetes mellitus (T2DM): A network meta-analysis (NMA). Diabetes. 2016;65:A566. <https://doi.org/10.2337/db16-2228-2366>

169. Luo Y, Bai R, Zhang W, Qin G. Selective sodium-glucose cotransporter-2 inhibitors in the improvement of hemoglobin and hematocrit in patients with type 2 diabetes mellitus: a network meta-analysis. Frontiers in Endocrinology. 2024;15. <https://doi.org/10.3389/fendo.2024.1333624>

170. Zaazouee MS, Hamdallah A, Helmy SK, Hasabo EA, Sayed AK, Gbreel MI, et al. Semaglutide for the treatment of type 2 Diabetes Mellitus: A systematic review and network meta-analysis of safety and efficacy outcomes. Diabetes and Metabolic Syndrome: Clinical Research and Reviews. 2022;16. <https://doi.org/10.1016/j.dsx.2022.102511>

171. Shyangdan DS, Uthman OA, Waugh N. SGLT-2 receptor inhibitors for treating patients with type 2 diabetes mellitus: A systematic review and network meta-analysis. BMJ Open. 2016;6. <https://doi.org/10.1136/bmjopen-2015-009417>

172. Bao Y, Hu Y, Shi M, Zhao Z. SGLT2 inhibitors reduce epicardial adipose tissue more than GLP-1 agonists or exercise interventions in patients with type 2 diabetes mellitus and/or obesity: A systematic review and network meta-analysis. Diabetes, Obesity and Metabolism. 2025;27:1096–112. <https://doi.org/10.1111/dom.16107>

173. Azharuddin M, Adil M, Ghosh P, Sharma M. Sodium-glucose cotransporter 2 inhibitors and fracture risk in patients with type 2 diabetes mellitus: A systematic literature review and Bayesian network meta-analysis of randomized controlled trials. Diabetes Research and Clinical Practice. 2018;146:180–90. <https://doi.org/10.1016/j.diabres.2018.10.019>

174. Palmer SC, Tendal B, Mustafa RA, Vandvik PO, Li S, Hao Q, et al. Sodium-glucose cotransporter protein-2 (SGLT-2) inhibitors and glucagon-like peptide-1 (GLP-1) receptor agonists for type 2 diabetes: Systematic review and network meta-analysis of randomised controlled trials. The BMJ. 2021;372. <https://doi.org/10.1136/bmj.m4573>

175. Lee SWH, Lee JY, Tan CSS, Wong CP. Strategies to make Ramadan fasting safer in type 2 diabetics: A systematic review and network meta-analysis of randomized controlled trials and observational studies. Medicine (United States). 2016;95. <https://doi.org/10.1097/MD.0000000000002457>

176. Karagiannis T, Malandris K, Avgerinos I, Stamati A, Kakotrichi P, Liakos A, et al. Subcutaneously administered tirzepatide vs semaglutide for adults with type 2 diabetes: a systematic review and network meta-analysis of randomised controlled trials. Diabetologia. 2024;67:1206–22. <https://doi.org/10.1007/s00125-024-06144-1>

177. Wu S, Cipriani A, Yang Z, Yang J, Cai T, Xu Y, et al. The cardiovascular effect of incretin-based therapies among type 2 diabetes: a systematic review and network meta-analysis. Expert Opinion on Drug Safety. 2018;17:243–9. <https://doi.org/10.1080/14740338.2018.1424826>

178. Yang J, Huang C, Wu S, Xu Y, Cai T, Chai S, et al. The effects of dipeptidyl peptidase-4 inhibitors on bone fracture among patients with type 2 diabetes mellitus: A network meta-analysis of randomized controlled trials. PLoS One. 2017;12. <https://doi.org/10.1371/journal.pone.0187537>

179. Wang J, Jin X, An P, Yu S, Mu Y. The Effects of Exenatide Once Weekly (EXQW) and Exenatide Twice a Day (EXBID) on Beta-Cell Function in Type 2 Diabetes: A Systematic Review and Network Meta-Analysis. Journal of Diabetes Research. 2019;2019. <https://doi.org/10.1155/2019/8083417>

180. Wu S, Gao L, Cipriani A, Huang Y, Yang Z, Yang J, et al. The effects of incretin-based therapies on β-cell function and insulin resistance in type 2 diabetes: A systematic review and network meta-analysis combining 360 trials. Diabetes, Obesity and Metabolism. 2019;21:975–83. <https://doi.org/10.1111/dom.13613>

181. Ling J, Cheng P, Ge L, Zhang DH, Shi AC, Tian JH, et al. The efficacy and safety of dipeptidyl peptidase-4 inhibitors for type 2 diabetes: a Bayesian network meta-analysis of 58 randomized controlled trials. Acta Diabetologica. 2019;56:249–72. <https://doi.org/10.1007/s00592-018-1222-z>

182. Alhindi Y, Avery A. The efficacy and safety of oral semaglutide for glycaemic management in adults with type 2 diabetes compared to subcutaneous semaglutide, placebo, and other GLP-1 RA comparators: A systematic review and network meta-analysis. Contemporary Clinical Trials Communications. 2022;28. <https://doi.org/10.1016/j.conctc.2022.100944>

183. Shen Y, Shi Q, Zou X, Meng W, Tian H, Du L, et al. Time-dependent risk of fracture in adults with type 2 diabetes receiving anti-diabetic drug: A one-stage network meta-analysis. Diabetes/Metabolism Research and Reviews. 2024;40. <https://doi.org/10.1002/dmrr.3780>

184. Lee CMY, Woodward M, Colagiuri S. Triple therapy combinations for the treatment of type 2 diabetes - A network meta-analysis. Diabetes Research and Clinical Practice. 2016;116:149–58. <https://doi.org/10.1016/j.diabres.2016.04.037>

185. Downes MJ, Bettington EK, Gunton JE, Turkstra E. Triple therapy in type 2 diabetes; A systematic review and network meta-analysis. PeerJ. 2015;2015. <https://doi.org/10.7717/peerj.1461>

**List of excluded studies and reasons**

**Non-type 2 diabetes (n=1)**

1. Zhao M, Sun S, Huang Z, Wang T, Tang H. Network meta-analysis of novel glucose-lowering drugs on risk of acute kidney injury. Clinical Journal of the American Society of Nephrology. 2021;16:70–8. <https://doi.org/10.2215/CJN.11220720>

**Non-SCI (n = 4)**

1. Fedyaeva VK, Ryzhov AO. Efficacy of dapagliflozin and empagliflozin for prevention of cardiovascular complications in patients with type 2 diabetes mellitus: a network meta-analysis. Medical Technologies Assessment and Choice. 2022;44:42–50. <https://doi.org/10.17116/medtech20224404142>

2. Fengqi L, Sanbao C, Houyu Z, Zuoxiang L, Shanshan W, Zhirong Y, et al. Sodium-glucose cotransporter 2 inhibitors and the risk of urinary tract infections or genital infections in adult patients with type 2 diabetes mellitus: a systematic review and network Meta-analysis of randomized controlled trials. Chinese Journal of Diabetes Mellitus. 2022;14:799–808. <https://doi.org/10.3760/cma.j.cn115791-20211217-00668>

3. Mannucci E, Torre E, Berto P. Dapagliflozin as add-on to metformin; network meta-analysis and budget impact analysis. Global and Regional Health Technology Assessment. 2015;2:125–34. <https://doi.org/10.5301/GRHTA.5000203>

4. Merész G, Szabó S, Dóczy V, Hölgyesi Á, Szakács Z. Relative frequency of urinary tract infections in patients affected by diabetes mellitus type 2 treated with metformin and SGLT2 inhibitor. Orvosi Hetilap. 2020;161:491–501. <https://doi.org/10.1556/650.2020.31690>

- **Non-NMA (n = 13)**

1. Khan Z, Naeem MO, Khan SK, Khan F, Abdullah M, Attique I, et al. Comparing Efficacy and Safety of Different Doses of Tirzepatide for the Treatment of Type 2 Diabetes Mellitus: a Meta-Analysis of Randomized Controlled Trials. Cureus. 2023;15:e44314. <https://doi.org/10.7759/cureus.44314>

2. Li D, Wang T, Shen S, Fang Z, Dong Y, Tang H. Urinary tract and genital infections in patients with type 2 diabetes treated with sodium-glucose co-transporter 2 inhibitors: A meta-analysis of randomized controlled trials. Diabetes, Obesity and Metabolism. 2017;19:348–55. <https://doi.org/10.1111/dom.12825>

3. Luo Y, Xia J, Zhao Z, Chang Y, Bee YM, Nguyen KT, et al. Effectiveness, safety, initial optimal dose, and optimal maintenance dose range of basal insulin regimens for type 2 diabetes: A systematic review with meta-analysis. Journal of Diabetes. 2023;15:419–35. <https://doi.org/10.1111/1753-0407.13381>

4. Malik AH, Yandrapalli S, Goldberg M, Jain D, Frishman WH, Aronow WS. Cardiovascular Outcomes With the Use of Sodium-Glucose Cotransporter-2 Inhibitors in Patients With Type 2 Diabetes and Chronic Kidney Disease: An Updated Meta-Analysis of Randomized Controlled Trials. Cardiology in Review. 2020;28:116–24. <https://doi.org/10.1097/CRD.0000000000000265>

5. Mima A, Gotoda H, Lee R, Murakami A, Akai R, Lee S. Effects of incretin-based therapeutic agents including tirzepatide on renal outcomes in patients with type 2 diabetes: A systemic review and meta-analysis. Metabolism open. 2023;17. <https://doi.org/10.1016/j.metop.2023.100236>

6. Orme M, Fenici P, Lomon ID, Wygant G, Townsend R, Roudaut M. A systematic review and mixed-treatment comparison of dapagliflozin with existing anti-diabetes treatments for those with type 2 diabetes mellitus inadequately controlled by sulfonylurea monotherapy. Diabetology and Metabolic Syndrome. 2014;6. <https://doi.org/10.1186/1758-5996-6-73>

7. Potts JE, Gray LJ, Brady EM, Khunti K, Davies MJ, Bodicoat DH. The effect of glucagon-like peptide 1 receptor agonists on weight loss in type 2 diabetes: A systematic review and mixed treatment comparison meta-analysis. PLoS One. 2015;10. <https://doi.org/10.1371/journal.pone.0126769>

8. Qiu M, Wei W, Wei XB, Liu SY. Updated network meta-analysis assessing the relative efficacy of 13 GLP-1 RA and SGLT2 inhibitor interventions on cardiorenal and mortality outcomes in type 2 diabetes. European Journal of Clinical Pharmacology. 2022;78:695–7. <https://doi.org/10.1007/s00228-021-03261-3>

9. Tang H, Dai Q, Shi W, Zhai S, Song Y, Han J. SGLT2 inhibitors and risk of cancer in type 2 diabetes: a systematic review and meta-analysis of randomised controlled trials. Diabetologia. 2017;60:1862–72. <https://doi.org/10.1007/s00125-017-4370-8>

10. Tang H, Fang Z, Wang T, Cui W, Zhai S, Song Y. Meta-Analysis of Effects of Sodium-Glucose Cotransporter 2 Inhibitors on Cardiovascular Outcomes and All-Cause Mortality Among Patients With Type 2 Diabetes Mellitus. American Journal of Cardiology. 2016;118:1774–80. <https://doi.org/10.1016/j.amjcard.2016.08.061>

11. Tang HL, Li DD, Zhang JJ, Hsu YH, Wang TS, Zhai SD, et al. Lack of evidence for a harmful effect of sodium-glucose co-transporter 2 (SGLT2) inhibitors on fracture risk among type 2 diabetes patients: a network and cumulative meta-analysis of randomized controlled trials. Diabetes, Obesity and Metabolism. 2016;18:1199–206. <https://doi.org/10.1111/dom.12742>

12. Tang H, Li D, Zhang J, Li Y, Wang T, Zhai S, et al. Sodium-glucose co-transporter-2 inhibitors and risk of adverse renal outcomes among patients with type 2 diabetes: A network and cumulative meta-analysis of randomized controlled trials. Diabetes, Obesity and Metabolism. 2017;19:1106–15. <https://doi.org/10.1111/dom.12917>

13. Willis M, Asseburg C, Neslusan C. Conducting and interpreting results of network meta-analyses in type 2 diabetes mellitus: A review of network meta-analyses that include sodium glucose co-transporter 2 inhibitors. Diabetes Research and Clinical Practice. 2019;148:222–33. <https://doi.org/10.1016/j.diabres.2019.01.005>

**Duplicate (n = 4)**

1. Ayesh H, Suhail S, Ayesh S, Niswender K. Comparative efficacy and safety of weekly dulaglutide versus weekly insulin in type 2 diabetes: a network meta-analysis of randomized clinical trials. Metabolism open. 2024;22:100284. <https://doi.org/10.1016/j.metop.2024.100284>

2. Kramer CK, Ye C, Campbell S, Retnakaran R. Comparison of New Glucose-Lowering Drugs on Risk of Heart Failure in Type 2 Diabetes: A Network Meta-Analysis. JACC Heart Fail. 2018;6:823–30. <https://doi.org/10.1016/j.jchf.2018.05.021>

3. Wang H, Zhang Q, Frois C, Vlajnic A, Wu E, Gerrits C, et al. Safety and efficacy of insulin glargine 300 U/mL (GLA-300) compared with other basal insulin therapies in patients with type 2 diabetes mellitus (T2DM)-a network meta-analysis (NMA). Diabetes. 2015;64:A26. <https://doi.org/10.2337/db151385>

4. Zhang J, Huan Y, Leibensperger M, Seo B, Song Y. Comparative Effects of Sodium-Glucose Cotransporter 2 Inhibitors on Serum Electrolyte Levels in Patients with Type 2 Diabetes: A Pairwise and Network Meta-Analysis of Randomized Controlled Trials. Kidney360. 2022;3:477–87. <https://doi.org/10.34067/kid.0006672021>

**Only abstract (n = 48)**

1. Acevedo JD, Lazcano G, Soto M, Castro A, Penaherrera C, Buitrago AF, et al. Cardiorenal Effects of Sglt2 Inhibitors and Finerenone in Type 2 Diabetes and Chronic Kidney Disease. Single, Dual or Triple Therapy? A Network Meta-Analysis. Circulation. 2022;146. <https://doi.org/10.1161/circ.146.suppl_1.10533>

2. Adil M, Ghosh P, Venkata SK, Raygude K, Gaba D, Kandhare AD, et al. Effect of anti-diabetic drugs on risk of fracture in type 2 diabetes mellitus patients: A network meta-analytic synthesis of randomized controlled trials of thiazolidinediones. Value in health. 2017;20:A526. <https://doi.org/10.1016/j.jval.2017.08.724>

3. Ahmad T, Adil M, Azharuddin M, Ansari S, Vohora D, Sharma M. Dipeptidyl Peptidase-4 Inhibitors and the Risk of Fractures in Type 2 Diabetes Mellitus Patients: A Bayesian Network Meta-Analysis. Curr Rev Clin Exp Pharmacol. 2025; <https://doi.org/10.2174/0127724328373328250624065307>

4. Avgerinos I, Malandris K, Stamati A, Liakos A, Kakaletsis N, Tsapas A, et al. Tirzepatide compared to subcutaneous semaglutide for type 2 diabetes: a network meta-analysis T. Karagiannis1. Diabetologia. 2023;66:S5. <https://doi.org/10.1007/s00125-023-05969-6>

5. Bacon T, Willis M, Johansen P, Neslusan C. Time until insulin initiation for canagliflozin (CANA) versus dapagliflozin (DAPA) in dual and triple therapy for type 2 diabetes mellitus (T2DM) in Ireland. Value in health. 2015;18:A55.

6. Barnett AH. Systematic review and network meta-analysis to compare dapagliflozin with other diabetes medications in combination with metformin for adults with type 2 diabetes. Therapeutic Research. 2016;37:398–9.

7. Bartmus T, Mansmann U. A systematic review and network meta-analysis assessing the effectiveness and tolerability of gliptins and sulfonylureas as monotherapy in patients with type 2 diabetes mellitus if metformin is not considered appropriate. Value in health. 2014;17:A333. <https://doi.org/10.1016/j.jval.2014.08.634>

8. Bekiari E, Karagiannis T, Avgerinos I, Liakos A, Malandris K, Manolopoulos A, et al. Glucose-lowering agents and incidence of amputation in patients with type 2 diabetes: a systematic review and network meta-analysis. Diabetologia. 2019;62:S458. <https://doi.org/10.1007/s00125-019-4946-6>

9. Dajani AI, Branko P. Essential phospholipids in the treatment of non-alcoholic fatty liver disease associated with type 2 diabetes or obesity: A systematic review and network meta-analysis. Hepatology International. 2020;14:S369–70. <https://doi.org/10.1007/s12072-020-10030-4>

10. Fadipe JJ. Efficacy of liraglutide compared to exenatide and insulin glargine in patients with diabetes type 2: A meta-analysis. Value in health. 2012;15:A467. <https://doi.org/10.1016/j.jval.2012.08.1504>

11. Ghosal S, Sinha B. WCN23-0081 SODIUM GLUCOSE CO-TRANSPORTER 2 INHIBITORS RANK HIGHER THAN RENIN-ANGIOTENSIN-ALDOSTERONE BLOCKERS AND FINERENONE IN PREVENTING END-STAGE-RENAL DISEASE IN PATIENTS WITH TYPE 2 DIABETES: A NETWORK META-ANALYSIS. Kidney International Reports. 2023;8: S187. <https://doi.org/10.1016/j.ekir.2023.02.417>

12. Goring SM, Huang T, Wygant G, Grishchenko M, Townsend R, Salsali A, et al. Efficacy of dapagliflozin compared with other oral antidiabetic agents added to metformin monotherapy among subjects with type 2 diabetes. Diabetologia. 2012;55:S305–6. <https://doi.org/10.1007/s00125-012-2688-9>

13. Hawkins N, Padhiar A, Thompson J, Scott DA, Eaton JN, Varol N, et al. Assessing consistency in a network meta-analysis to compare once weekly dulaglutide versus other GLP-1 receptor agonists in patients with type 2 diabetes. Value in health. 2014;17: A335. <https://doi.org/10.1016/j.jval.2014.08.641>

14. Hussein H, Zaccardi F, Khunti K, Davies MJ, Patsko E, Kloecker D, et al. Efficacy and safety of sodium-glucose co-transporter-2inhibitorsandglucagon-like peptide-1 receptor agonists: A systematic review and network meta-analyses. Diabetic medicine. 2020; 37:178. <https://doi.org/10.1111/dme.14245>

15. Jammah A. Safety and efficacy of insulin glargine/ lixisenatide and insulin degludec/insulin aspart in type 2 diabetes patients not controlled on basal insulin: A network meta-analysis. Diabetes Technology and Therapeutics. 2020;22:A185–6. <https://doi.org/10.1089/dia.2020.2525.abstracts>

16. Jindal R, Gupta J, Arora A, Kaur M, Kumar R, Kaushik P. Network meta-analysis of fixed dose combination therapies for the first-line treatment of type 2 diabetes mellitus. Value in health. 2012;15: A660. <https://doi.org/10.1016/j.jval.2012.08.339>

17. Kodama S, Sato T, Yamada MH, Yamamoto M, Matsubayashi Y, Ishiguro H, et al. Network Meta-analysis of Hypoglycemic Treatment Regimens with Potential Risk of Hypoglycemia in Terms of Glycemic Control and Severe Hypoglycemia Diabetes. 2022;71. <https://doi.org/10.2337/db22-378-P>

18. Li Y, Murray E, Del Aguila M, Cui N, Wen Y. Network meta-analysis of insulin effectiveness among basal insulins in type 2 diabetes mellitus. Diabetes/Metabolism Research and Reviews. 2018;34:20. <https://doi.org/10.1002/dmrr.3079>

19. Liu C. Comparative efficacy of anti-diabetic agents on nonalcoholic fatty liver disease in patients with type 2 diabetes: A networkmeta-analysis. Diabetologia. 2020;63:S416–7. <https://doi.org/10.1007/s00125-020-05221-5>

20. Lozano-Ortega G, Goring S, Bennett H, Sternhufvud C, Mukherjee J, Bergenheim K. Network meta-analysis of treatments for type 2 diabetes mellitus following failure with metformin + sulfonylurea. Diabetes. 2015;64: A329. <https://doi.org/10.2337/db159321471>

21. Men P, Qu S, Luo W, Liu Y, Li C, Zhai S. Comparative Efficacy and Safety of Lixisenatide and Insulin Regimens in the Treatment of Patients with Type 2 Diabetes Mellitus Inadequately Controlled by Basal Insulin: A Systematic Review and Network Meta-Analysis. Value in health. 2018;21: S35. <https://doi.org/10.1016/j.jval.2018.07.269>

22. Orme ME, Bell K, Dhankhar P. Network meta-analysis of diabetes drug classes as add-on to metformin for T2D. Diabetes. 2015;64: A310. <https://doi.org/10.2337/db159321471>

23. Orme ME, Fenici P, Duprat Lomon I, Wygant G, Townsend R, Roudaut M. A systematic review and network meta-analysis of second-line anti-diabetes treatments for those with type 2 diabetes mellitus inadequately controlled by sulfonylurea monotherapy. Value in health. 2013;16: A432. <https://doi.org/10.1016/j.jval.2013.08.629>

24. Pacou M, Taieb V, Abrams KR, Diels J, Van Sanden S, Garg M, et al. Bayesian network meta-analysis to assess relative efficacy and safety of canagliflozin in patients with type 2 diabetes mellitus (T2DM) inadequately controlled with metformin. Value in health. 2013;16: A 609. <https://doi.org/10.1016/j.jval.2013.08.1747>

25. Pacou M, Taieb V, Abrams KR, Diels J, Van Sanden S, Garg M, et al. Bayesian network meta-analysis to assess the relative efficacy and safety of canagliflozin in patients with type 2 diabetes mellitus (T2DM) inadequately controlled on metformin and sulphonylurea (MET+SU). Value in health. 2013;16: A431. <https://doi.org/10.1016/j.jval.2013.08.623>

26. Palmer S, Mavridis D, Nicolucci A, Craig J, Tonelli M, Johnson D, et al. Glucose-lowering drugs added to existing therapies and risks of mortality and cardiovascular disease in type 2 diabetes: network meta-analysis of randomized trials. Nephrology dialysis transplantation. 2017;32:iii264‐iii265. <https://doi.org/10.1093/ndt/gfx149>

27. Remonti LR, Dias S, Leitão CB, Kramer CK, Klassmann LP, Welton N, et al. Classes of antihypertensive agents and mortality in hypertensive patients with type 2 diabetes-network meta-analysis. Diabetes. 2013;62:A111. <https://doi.org/10.2337/db13-388-679>

28. Schroeder M, Taieb V, Belhadi D, Seyla-Hammer C, Hemels M, Nielsen AT. Bayesian network meta-analysis (NMA) to assess the relative efficacy of canagliflozin monotherapy over 26 weeks in patients with type 2 diabetes mellitus (T2DM). Value in health. 2015;18:A56.

29. Schroeder M, Taieb V, Pacou M, Ho S, Nielsen AT, Schubert A, et al. A network meta-analysis to assess options for treatment intensification for patients with type 2 diabetes inadequately controlled on dual therapy. Diabetologia. 2015;58: S347. <https://doi.org/10.1007/s00125-015-3687-4>

30. Scott D, Boye KS, Timlin L, Best JH, Clark J. A network meta analysis to compare glycaemic control in patients with type 2 diabetes treated with exenatide once weekly or liraglutide. Value in health. 2011;14:A472–3. <https://doi.org/10.1016/j.jval.2011.08.1311>

31. Sethi Y, Muhseenah, Fernandez B, Rani I, Rahna K, Shamim MA, et al. ONE DRUG FOR MULTIMORBID HYPERTENSION AND TYPE 2 DIABETES? A DOSE-WISE NETWORK META-ANALYSIS OF TIRZEPATIDE. Journal of the American College of Cardiology. 2024; 83:1732. <https://doi.org/10.1016/S0735-1097(24)03722-7>

32. Shanshan W, Jun Y, Ting C, Zhirong Y, Yuan Z, Sanbao C, et al. Effect of DPP-4 inhibitors on body weight in type 2 diabetes: A systematic review and network meta-analysis. Diabetes/Metabolism Research and Reviews. 2016; 32:53. <https://doi.org/10.1002/dmrr.2856>

33. Stevens JW, Harvey RC, Johnson M, Khunti K. Preventing the progression to type 2 diabetes mellitus in adults at high risk: A systematic review and network meta-analysis of lifestyle, pharmacological and surgical interventions. Value in health. 2014;17: A335. <https://doi.org/10.1016/j.jval.2014.08.643>

34. Sun F, Wu S, Chai S, Yang Z, Yu K, Zhan S. Impact of GLP-1RA on heart rate , blood pressure and hypertension among type 2 diabetes: A systematic review and network meta-analysis. Value in health. 2014;17:A719–20. <https://doi.org/10.1016/j.jval.2014.08.010>

35. Sun F, Yu K, Wu S, Zhang Y, Yang Z, Ji L, et al. Gastrointestinal adverse events of glucagon-like peptide-1 receptor agonists for type 2 diabetes mellitus: A pairwise and network meta-analysis. Pharmacoepidemiology and Drug Safety. 2013;22:72–3. <https://doi.org/10.1002/pds.3512>

36. Taieb V, Pacou M, Schroeder M, Nielsen AT, Neslusan C, Schubert A. Bayesian network meta-analysis (NMA) to assess the relative efficacy of canagliflozin in patients with type 2 diabetes mellitus (T2DM) inadequately controlled with insulin. Value in health. 2015;18: A598.

37. Tang H, Dai Q, Shi W, Zhai S, Song Y, Han J. Sodium-glucose cotransporter 2 inhibitors and risk of cancer in patients with type 2 diabetes: A network meta-analysis of randomized controlled trials. Pharmacoepidemiology and Drug Safety. 2017;26:396–7. <https://doi.org/10.1002/pds.4275>

38. Tang H, Dai Q, Zhang J, Song Y. Comparative effects of sodium-glucose cotransporter 2 inhibitors on serum magnesium levels in patients with type 2 diabetes: A network meta-analysis of randomized controlled trials. Magnesium Research. 2019;32:100–1. <https://doi.org/10.1684/mrh.2020.0459>

39. Tang H, Dai Q, Zhang J, Song Y. Comparative effects of sodiumglucose cotransporter 2 inhibitors on serum electrolyte levels in patients with type 2 diabetes: A network meta-analysis of randomized controlled trials. Pharmacoepidemiology and Drug Safety. 2019; 28:566. <https://doi.org/10.1002/pds.4864>

40. Tang H, Xia J, Yang K. Comparative effectiveness of long-term lifestyle and pharmacological interventions for primary prevention of type 2 diabetes: A Network meta-analysis of randomized controlled trials. Circulation. 2019;139. <https://doi.org/10.1161/circ.139.suppl_1.035>

41. Thorlund K, Siliman G, Eapen S, Lund S, Palencia R. Comparative efficacy and safety of empagliflozin with other antidiabetic drugs for the third line treatment of type 2 diabetes mellitus. Value in health. 2014;17: A333. <https://doi.org/10.1016/j.jval.2014.08.630>

42. Tsapas A, Avgerinos I, Karagiannis T, Malandris K, Andreadis P, Manolopoulos A, et al. Comparative efficacy and safety of medications for type 2 diabetes: a comprehensive systematic review and network meta-analysis. Diabetologia. 2019;62: S429. <https://doi.org/10.1007/s00125-019-4946-6>

43. Tsapas A, Madenidou AV, Karagiannis T, Paschos P, Athanasiadou E, Liakos A, et al. Basal insulin analogues for type 2 diabetes: Systematic review and network meta-analysis. Diabetologia. 2016;59: S403. <https://doi.org/10.1007/s00125-016-4046-9>

44. Van Sanden S, Diels J, Guillon P, Nielsen AT. Bayesian network meta-analysis (NMA) to assess relative efficacy of canagliflozin (CANA) versus glucagon-like peptide-1 (GLP-1) agonists in dual and triple therapy in patients with type 2 diabetes mellitus (T2DM). Value in health. 2015;18: A54.

45. Wu S, Yang J, Cai T, Xu Y, Yang Z, Zhang Y, et al. Cardiovascular effects of incretin-based therapies in patients with type 2 diabetes: A systematic review and network meta-analysis. The Lancet Diabetes and Endocrinology. 2016;4:S18. <https://doi.org/10.1016/S2213-8587(16)30373-4>

46. Yang J, Wu S, Xu Y, Cai T, Yang Z, Zhan S, et al. Impact of dipeptidyl peptidase-4 inhibitors on bone fracture among patients with type 2 diabetes mellitus: A network meta-analysis. Diabetes/Metabolism Research and Reviews. 2016;32:4–5. <https://doi.org/10.1002/dmrr.2854>

47. Yoon J, Hahn S, Min SH, Cho YM, Moon SJ. Network meta-analysis of antidiabetic treatments added to insulin therapy in patients with type 2 diabetes mellitus. Value in health. 2016;19: A666.

48. Zheng S, Roddick A. Acute coronary syndrome in type 2 diabetes treated using SGLT-2 inhibitors, GLP-1 agonists and DPP-4 inhibitors: A comparative network meta-analysis of 127,731 individuals. Heart. 2018;104:A59–60. <https://doi.org/10.1136/heartjnl-2018-BCS.66>

**Ineligible intervention (n = 12)**

1. Allam AR, Helal MB, Alhateem MS, Shehab MA, Elshaar AG, Saeda MA, et al. Network meta-analysis of randomized control trials evaluating the effectiveness of various probiotic formulations in patients with type 2 diabetes mellitus. Diabetology and Metabolic Syndrome. 2025;17. <https://doi.org/10.1186/s13098-025-01841-2>

2. Cui JY, Zhou RR, Han S, Wang TS, Wang LQ, Xie XH. Statin therapy on glycemic control in type 2 diabetic patients: A network meta-analysis. Journal of Clinical Pharmacy and Therapeutics. 2018;43:556–70. <https://doi.org/10.1111/jcpt.12690>

3. Hu YB, Hu ED, Fu RQ. Statin use and cancer incidence in patients with type 2 diabetes mellitus: A network meta-analysis. Gastroenterology Research and Practice. 2018;2018. <https://doi.org/10.1155/2018/8620682>

4. Juhász AE, Greff D, Teutsch B, Gede N, Hegyi P, Horváth EM, et al. Galactomannans are the most effective soluble dietary fibers in type 2 diabetes: a systematic review and network meta-analysis. American Journal of Clinical Nutrition. 2023;117:266–77. <https://doi.org/10.1016/j.ajcnut.2022.12.015>

5. Kazemi A, Ryul Shim S, Jamali N, Hassanzadeh-Rostami Z, Soltani S, Sasani N, et al. Comparison of nutritional supplements for glycemic control in type 2 diabetes: A systematic review and network meta-analysis of randomized trials. Diabetes Research and Clinical Practice. 2022;191. <https://doi.org/10.1016/j.diabres.2022.110037>

6. Lu Z, Zhang X, Xie Y. Regulatory patterns of Chinese patent medicine for lipid metabolism disorders in patients with type 2 diabetes mellitus complicated by ischemic stroke: A systematic review and network meta-analysis. Medicine (United States). 2024;103:E35050. <https://doi.org/10.1097/MD.0000000000035050>

7. Ojo O, Wang X, Ojo OO, Brooke J, Jiang Y, Dong Q, et al. The Effect of Prebiotics and Oral Anti-Diabetic Agents on Gut Microbiome in Patients with Type 2 Diabetes: A Systematic Review and Network Meta-Analysis of Randomised Controlled Trials. Nutrients. 2022;14. <https://doi.org/10.3390/nu14235139>

8. Pang B, Zhao LH, Li XL, Song J, Li QW, Liao X, et al. Different intervention strategies for preventing type 2 diabetes mellitus in China: A systematic review and network meta-analysis of randomized controlled trials. Diabetes, Obesity and Metabolism. 2018;20:718–22. <https://doi.org/10.1111/dom.13121>

9. Pitak P, Boonpattharatthiti K, Fuangchan A, Krass I, Dhippayom T. How can we best support insulin self-titration in type 2 diabetes patients: A systematic review and network meta-analysis. Diabetes and Metabolic Syndrome: Clinical Research and Reviews. 2025;19. <https://doi.org/10.1016/j.dsx.2025.103221>

10. Vejakama P, Thakkinstian A, Lertrattananon D, Ingsathit A, Ngarmukos C, Attia J. Reno-protective effects of renin-angiotensin system blockade in type 2 diabetic patients: A systematic review and network meta-analysis. Diabetologia. 2012;55:566–78. <https://doi.org/10.1007/s00125-011-2398-8>

11. Xie XX, Liu P, Wan FY, Lin SG, Zhong WL, Yuan ZK, et al. Blood pressure lowering and stroke events in type 2 diabetes: A network meta-analysis of randomized controlled trials. International Journal of Cardiology. 2016;208:141–6. <https://doi.org/10.1016/j.ijcard.2016.01.197>

12. Yang Q, Zheng R, Wang S, Zhu J, Li M, Wang T, et al. Systolic Blood Pressure Control Targets to Prevent Major Cardiovascular Events and Death in Patients with Type 2 Diabetes: A Systematic Review and Network Meta-Analysis. Hypertension. 2023;80:1640–53. <https://doi.org/10.1161/HYPERTENSIONAHA.123.20954>

**Not evaluating(n = 2)**

1. Goring S, Hawkins N, Wygant G, Roudaut M, Townsend R, Wood I, et al. Dapagliflozin compared with other oral anti-diabetes treatments when added to metformin monotherapy: A systematic review and network meta-analysis. Diabetes, Obesity and Metabolism. 2014;16:433–42. <https://doi.org/10.1111/dom.12239>

2. Webb N, Orme M, Witkowski M, Nakanishi R, Langer J. A Network Meta-Analysis Comparing Semaglutide Once-Weekly with Other GLP-1 Receptor Agonists in Japanese Patients with Type 2 Diabetes. Diabetes therapy. 2018;9:973–86. <https://doi.org/10.1007/s13300-018-0397-1>
